# BMC *Genomics* reviewer acknowledgement 2015

**DOI:** 10.1186/s12864-016-2451-6

**Published:** 2016-03-02

**Authors:** Derek Anane

**Affiliations:** BioMed Central, Floor 6, 236 Gray’s Inn Road, London, WC1X 8HB UK

## Abstract

ᅟ

**Philippos A Papathanos**

Italy

**Reidunn Aalen**

Norway

**Alejandro Aballay**

USA

**Zakaria Abd Elmageed**

USA

**Amy Abdulovic-Cui**

USA

**Ikuro Abe**

Japan

**José A Abelenda**

Netherlands

**Jason Abernathy**

USA

**Wolf-Rainer Abraham**

Germany

**Josep F Abril**

Spain

**Serena Aceto**

Italy

**Nathalie Acevedo**

Sweden

**Guillaume Achaz**

France

**Kaia Achim**

Germany

**Wafa Achouak**

France

**Hervé Acloque**

France

**Shelley Adamo**

Canada

**Charles Addo-Quaye**

USA

**David Adelson**

Australia

**Adeolu Adewoye**

UK

**Andrew Adey**

USA

**Bishawo Adhikari**

USA

**Daniel Adkins**

USA

**Evelien Adriaenssens**

South Africa

**Toni Aebischer**

Germany

**Claudio Afonso**

USA

**Fabian Afonso-Grunz**

Germany

**Pinky Agarwal**

India

**Manu Agarwal**

India

**Sam Aggrey**

USA

**Lucymara F Agnez-Lima**

Brazil

**Ainhoa Agorreta**

Spain

**Pankaj B Agrawal**

USA

**Derek Aguiar**

USA

**Ruben Aguilar**

USA

**Nathan Ahlgren**

USA

**Jaegyoon Ahn**

USA

**Elizabeth Ainsworth**

USA

**Steven D. Aird**

Japan

**Karen Aitken**

Australia

**Timothy Aitman**

UK

**Omar Akbari**

USA

**Schahram Akbarian**

USA

**Guray Akdogan**

Turkey

**Nobuyoshi Akimitsu**

Japan

**Ibrahim Alabdulkareem**

Saudi Arabia

**Mar Albà**

Spain

**Frank Albert**

USA

**Giles Albert**

France

**Jennifer Alcaino**

Chile

**Pedro Alcolea**

Spain

**Yuriy Alekseyev**

USA

**Andrey Alexeyenko**

Sweden

**Jessica Alfoldi**

USA

**Sher Ali**

India

**Pietro Alifano**

Italy

**Heather Allison**

UK

**Jens Allmer**

Turkey

**David Allred**

USA

**Nalvo Almeida**

USA

**Juan Alonso**

Spain

**Sam Alsford**

UK

**Manessha Aluru**

USA

**Carlos Alvarez**

USA

**Sergio Alvarez**

Chile

**Santiago Alvarez Prado**

Argentina

**Karen Alviar**

Philippines

**Elisabete Amaral**

Brazil

**Mónica Amblar**

Spain

**Marcel Amichot**

France

**Marcel Amills**

Spain

**Keenan Amundsen**

USA

**Yong-Qiang An**

USA

**Hong An**

China

**Guruprasad Ananda**

USA

**Sirinart Ananvoranich**

Canada

**Satyanarayana Ande**

USA

**Claus Andersen**

Denmark

**Jonathon Anderson**

Australia

**Bjorn Andersson**

Sweden

**Leif Andersson**

Sweden

**Martin Andersson**

Sweden

**Erik Andreasson**

Sweden

**Ólafur Andrésson**

Iceland

**Toby Andrew**

UK

**Ching-Seng Ang**

Australia

**Peter Antal**

Hungary

**De Tomaso Anthony**

USA

**Peter Antinozzi**

USA

**Jun-Ya Aoki**

Japan

**Ruslan Aphasizhev**

USA

**Gayathri Arakere**

India

**Armando Aranda-Anzaldo**

Mexico

**Susana Araújo**

Portugal

**Bruno Arcà**

Italy

**Alan L. Archibald**

UK

**Kazim Yalcin Arga**

Turkey

**Anagnostis Argiriou**

Greece

**Dan Arking**

USA

**Peter Armbruster**

USA

**Gemma Armengol**

Spain

**John Armour**

UK

**Irena Artamonova**

Russian Federation

**Adam Arterbery**

USA

**Neelakantan Arumugam**

India

**Pere Arus**

Spain

**Lars Arvestad**

Sweden

**Philip Ashton**

UK

**Mehar Hasan Asif**

India

**Yassen Assenov**

Germany

**Raquel Assis**

USA

**Watanabe Atsushi**

Japan

**Marcella Attimonelli**

Italy

**Kin Fai Au**

USA

**Scott Auerbach**

USA

**Charles Auffray**

France

**Ganley Austen**

New Zealand

**Ingo Birger Autenrieth**

Germany

**Javier Avalos**

Spain

**Komlan Avia**

France

**Michael Axtell**

USA

**Katie Ayers**

Australia

**A Aykut**

Turkey

**Frank Aylward**

USA

**Nadia Ayoub**

USA

**Brian Ayre**

USA

**Julien Ayroles**

USA

**Vasco Azevedo**

Brazil

**Majid Azizi**

Iran

**Tomas Babak**

USA

**Igor Babiak**

Norway

**Lutz Bachmann**

Norway

**Alexander Bachmanov**

USA

**Ekaterina D. Badaeva**

Russian Federation

**Bouabid Badaoui**

Italy

**Dayakar Badri**

USA

**Melinda Baerwald**

USA

**Christine Baes**

Canada

**Kristin Baetz**

Canada

**Paramjeet Bagga**

USA

**Alessandro Bagnato**

Italy

**Swneke D. Bailey**

Canada

**Harsh Bais**

USA

**Soren Bak**

Denmark

**Richard Baker**

USA

**Michelle Baker**

Australia

**Scott E. Baker**

USA

**Guus Bakkeren**

Canada

**Rama Balakrishnan**

USA

**Vicente Balanzà**

Spain

**Ana-Rosa Ballester**

Spain

**Brunilda Balliu**

USA

**Mehmat Baloglu**

Turkey

**Ian Bancroft**

UK

**Claudio Bandi**

Italy

**Nirmalya Bandyopadhyay**

USA

**Purnima Bandyopadhyay**

USA

**John Bannantine**

USA

**Yan-Yuan Bao**

China

**Fernando Baquero**

Spain

**Elena Baraldi**

Italy

**Daniel Barbash**

USA

**Nuno L Barbosa-Morais**

Portugal

**Gianni Barcaccia**

Italy

**Amelie Bardil**

Switzerland

**William Barendse**

Australia

**Luca Bargelloni**

Italy

**Jamie L. Barger**

USA

**Andrew Barnes**

Australia

**Amalia Barone**

Italy

**Rodolphe Barrangou**

USA

**Philippe Barre**

France

**Richárd Bártfai**

Netherlands

**Jerome Bartholome**

France

**Daniella Bartholomeu**

Brazil

**Surajit Basak**

India

**Susan Baserga**

USA

**Chris Bass**

UK

**Nahla Bassil**

USA

**John Bastiaansen**

Netherlands

**Patrick Bastien**

France

**Chhandak Basu**

USA

**Philip Bates**

USA

**Oliver Batistic**

Germany

**Jacqueline Batley**

Australia

**Jyotsna Batra**

Australia

**Jacques Batut**

France

**Denis Carolin Jessica Bauer**

Australia

**Paul Baum**

USA

**Andrew Beacham**

UK

**Paul Beare**

USA

**Philippe Beaujard**

France

**Luis Augusto Becerra Lopez-Lavalle**

Colombia

**Catherina G. Becker**

UK

**Jörg Becker**

Portugal

**Rasheedunnisa Begum**

India

**Tim Beissinger**

USA

**Stefan Bekiranov**

USA

**Brendan Bell**

Canada

**Jayakumar Belli Kullan**

Italy

**John Belote**

USA

**Robert Belshaw**

UK

**Fraǹçois Belzile**

Canada

**David Benjamin**

USA

**Bernhard Benkel**

Canada

**Joshua Benoit**

USA

**Touati Benoukraf**

Singapore

**Martin Bens**

Germany

**Jacqueline Benson**

USA

**Andrew Bent**

USA

**Stephen Bentley**

UK

**Jagadish Bentur**

India

**Beatrix Berdal**

UK

**Dave Berger**

South Africa

**Jamila Bernardi**

Italy

**Matthias Bernt**

Germany

**Andrea Betancourt**

Austria

**Debashish Bhattacharya**

USA

**Christopher Bidwell**

USA

**Matthias Bieg**

Germany

**Douglas Bielenberg**

USA

**Holly Bik**

UK

**Tugce Bilgin Sonay**

Switzerland

**Gerald F. Bills**

USA

**K Bilyeu**

USA

**Terry Bird**

USA

**Karen Bishop**

New Zealand

**Kimberly Bishop-Lilly**

USA

**Wilbert Bitter**

Netherlands

**Teshome Bizuayehu**

Norway

**Michael Black**

Australia

**Damer Blake**

UK

**Jeffrey Blanchard**

USA

**Stephanie Blandin**

France

**Giovanni Blandino**

Italy

**Anne Bleicher**

Germany

**Tugce Bligin Sonay**

Switzerland

**Anders Blomberg**

Sweden

**Paul Blum**

USA

**Miroslav Blumenberg**

USA

**Julien Bobe**

France

**Christoph Bock**

Germany

**Helge Bode**

Germany

**Judith Boer**

Netherlands

**Paul Boersema**

Switzerland

**Dario Boffelli**

USA

**Blaise Boles**

USA

**Alexander Bolshoy**

Israel

**Sebastain Boltana**

Chile

**Aureliano Bombarely**

USA

**Ursula Bond**

Ireland

**Paola Bonfante**

Italy

**German Bonilla-Rosso**

Sweden

**Hidemasa Bono**

Japan

**Jeremy Bono**

USA

**Marije Booman**

Canada

**Stephanie Booth**

Canada

**Glen Borchert**

USA

**Russell Borski**

USA

**Nicole Borth**

Austria

**Kirill Borziak**

USA

**Emanuele Bosi**

Italy

**Daniela Bosisio**

Italy

**Janine Bosse**

UK

**Krassimira Botcheva**

USA

**Natasha Botwright**

Australia

**Jeremie Boucher**

Sweden

**Jean-Paul Bouchet**

France

**Aniek Bouwman**

Netherlands

**Henk Bovenhuis**

Netherlands

**Nathan Bowen**

USA

**Jennifer Bowen**

USA

**Walter Boyce**

USA

**Jon Boyle**

USA

**Jan Brabek**

Czech Republic

**Valerie Bracchi-Ricard**

USA

**Christopher Bradburne**

USA

**Dan Bradley**

Ireland

**Cornelia Braicu**

ROMANIA

**Gerhard Braus**

Germany

**Kelly Brayton**

USA

**A Braza-Boils**

Spain

**Felix Breden**

Canada

**Phil Bregitzer**

USA

**Klaus Brehm**

Germany

**Jacco Briede**

Netherlands

**Marine Brieuc**

USA

**Karina Brinkrolf**

Germany

**Susanne Brix**

Denmark

**Matthias Brock**

UK

**Peter Bron**

Netherlands

**Roland Brosch**

France

**C. Titus Brown**

USA

**Susan Brown**

USA

**Kathleen Brown**

USA

**Jay Brown**

USA

**Gegory Brown**

Canada

**James Brown**

USA

**Steven Brown**

USA

**John Browne**

Ireland

**Glenn Browning**

Australia

**Claude Bruand**

France

**James Brugarolas**

USA

**Harry Brumer**

Canada

**Søren Brunak**

Denmark

**Frederic Brunet**

France

**A Brüniche-Olsen**

Australia

**Giovanni Bubici**

Italy

**David Buchner**

USA

**Micheal Buck**

USA

**Maria Buerstmayr**

Austria

**Lee Bulla**

USA

**Laurel Burall**

USA

**Daniel Burckhardt**

Switzerland

**Tilmann Bürckstümmer**

USA

**Diane Burgess**

USA

**Alexandrina Burlacu**

ROMANIA

**Thorsten Burmester**

Germany

**Pierre Bushel**

USA

**Matthew Butchbach**

USA

**Matteo Buti**

Italy

**Teresia Buza**

USA

**Leonid Bystrykh**

Netherlands

**Michella C. Barton**

USA

**Zameel Cader**

UK

**Hongmei Cai**

China

**Francesco Calabrese**

Italy

**Michael Calcutt**

USA

**Isabelle Caldelari**

France

**Federica Calevro**

France

**Tercilio Calsa Junior**

Brazil

**Juan Calvete**

Spain

**Veronica Cambiazo**

Chile

**Daniel Camerini-Otero**

USA

**Nzali Campbell**

USA

**Tony Campillo**

France

**Jose Campos**

UK

**Victoria Campuzano**

Spain

**Adelino Canario**

Portugal

**Cristian Cañestro**

Spain

**Cinzia Cantacessi**

UK

**Brandi Cantarel**

USA

**Dario Cantu**

USA

**Tobias Cantz**

Germany

**Guojie Cao**

USA

**Huansheng Cao**

USA

**Weiguo Cao**

USA

**S Cao**

China

**Jiashu Cao**

China

**Blanche Capel**

USA

**Antonio Carapelli**

Italy

**Lucia Carbone**

USA

**Mari Francesca Cardone**

Italy

**Karen Carleton**

USA

**John Carman**

USA

**Francisco Javier Carmona Sanz**

USA

**Steve Carr**

Canada

**Enrique Carrillo**

Spain

**Ronan Carroll**

USA

**Clay Carter**

USA

**Marco Caruso**

Italy

**Luciano Cascione**

Switzerland

**David Casero**

USA

**Benoit Castandet**

USA

**Raúl Castanera**

Spain

**Luisa Castiblanco**

USA

**Fidel Ovidio Castro**

Chile

**Juan Carlos Castro Gómez**

PERU

**Andrea Cavallini**

Italy

**Brad Cavinder**

USA

**Francisco Cazorla**

Spain

**Alberto Cenci**

France

**Gustavo Cerqueira**

USA

**Margarita Cervera**

Spain

**David Chagne**

New Zealand

**Domitille Chalopin**

USA

**Srikar Chamala**

USA

**Tingfung Chan**

Hong Kong

**Kok-Gan Chan**

Malaysia

**Mark Chance**

USA

**Wen-Chi Chang**

Taiwan

**Wen-Chi Chang**

Taiwan

**S. B. Chang**

Taiwan

**Yanjie Chao**

Germany

**Brad Chapman**

USA

**Robert Chapman**

USA

**Mark Chapman**

UK

**Carole Charlier**

Belgium

**Gilles Charmet**

France

**Pep Charusanti**

USA

**Christophe Chassard**

Switzerland

**John Chaston**

USA

**Aniruddha Chatterjee**

New Zealand

**Roy Chaudhuri**

UK

**Vinita Chauhan**

Canada

**Harsh Chauhan**

India

**Rajinder S. Chauhan**

India

**Cedric Chauve**

Canada

**Ian Cheeseman**

USA

**Chia-Ying Chen**

Taiwan

**Senyu Chen**

USA

**Luke Chen**

USA

**Zigui Chen**

USA

**Zhi Chen**

Finland

**Yu Tin Chen**

Taiwan

**Ming Chen**

China

**Rui Chen**

USA

**Yi Ywan Chen**

Taiwan

**Jin Chen**

USA

**Bin Chen**

China

**Min Chen**

Australia

**Li-Song Chen**

China

**Qijun Chen**

China

**Xin Chen**

USA

**Yuan-Tsong Chen**

Taiwan

**Michelle Y.Q. Chen**

China

**Yoasheng Chen**

China

**Hong Chen**

China

**Fure-Chyi Chen**

Taiwan

**Jingjing Chen**

China

**Shilin Chen**

China

**Chien-Yu Chen**

Taiwan

**Jun Chen**

China

**Wei-Chung Cheng**

Taiwan

**Wing Cheung**

Canada

**Peter Cheung**

China

**Ching-Lung Cheung**

Hong Kong

**A Chevre**

France

**Sung Wook Chi**

South Korea

**Yu-Chung Chiang**

Taiwan

**Tzen-Yuh Chiang**

Taiwan

**Yu-Chung Chiang**

Taiwan

**Chai Ching**

Japan

**Patrick Chinnery**

UK

**Ioana Chintauan-Marquier**

France

**Chi-Chou Chiu**

Taiwan

**Sung Won Cho**

South Korea

**Won Kyong Cho**

North Korea

**Jung-Woo Choi**

South Korea

**Dongwoog Choi**

South Korea

**Amanda Chong**

UK

**Heng Chooi**

Australia

**Wilaiwan Chotigeat**

Thailand

**Wen-Chi Chou**

USA

**Anuradha Chowdhary**

India

**Henrik Christensen**

Denmark

**Alan C. Christensen**

USA

**Zhaoqing Chu**

China

**Yen-Wei Chu**

Taiwan

**Trees-Juen Chuang**

Taiwan

**Meichu Chung**

Taiwan

**Ulas Cinar**

USA

**Daniel Ciobanu**

USA

**Giovanni Ciriello**

USA

**Elizabeth Cirulli**

USA

**Jérome Clain**

France

**Leigh Clark**

USA

**Matthew Clark**

USA

**Clifford Clark**

Canada

**Melody Susan Clark**

UK

**Neil Clarke**

Singapore

**M. Gonzalo Claros**

Spain

**Christine Clayton**

Germany

**David Clayton**

UK

**Jackie Cliff**

UK

**Martin Clift**

Switzerland

**Timothy Close**

USA

**Marién Cobo**

Spain

**Sergio Cocozza**

Italy

**Julie Cocquet**

France

**Christopher Coe**

USA

**Adriano Coelho**

Brazil

**Ian Colditz**

Australia

**Shelley Cole**

USA

**Stephen Coleman**

USA

**Justine Collier**

Switzerland

**Maria Colome-Tatche**

Netherlands

**Inaki Comas**

Spain

**Gavin Conant**

USA

**Ciaran Condon**

France

**Yongzheng Cong**

USA

**Leonardo Congiu**

Italy

**Erin Conlon**

USA

**Ian Connerton**

UK

**Erin Connor**

USA

**Kwasi Connor**

USA

**Matthew Conte**

USA

**Lydia Contreras**

USA

**Thomas Conway**

Australia

**Vanessa Corby-Harris**

USA

**Mathilde Cordellier**

Germany

**Pierre Cornelis**

Belgium

**Emmanuel Cornillot**

France

**Miguel Corona**

USA

**Nicolas Corradi**

Canada

**Mireia Coscolla**

Spain

**Marco Cosentino Lagomarsino**

France

**Maria Costa**

Portugal

**Maria Costantini**

Italy

**Nicole Cotte-Pattat**

France

**David Cotter**

Ireland

**Juan Pablo Coulleri**

Argentina

**Christine Coustau**

France

**Luiz Lehmann Coutinho**

Brazil

**Felipe Coutinho**

Brazil

**Gordon Cramb**

UK

**Robert Cramer**

USA

**Orwald Crasta**

USA

**Kelly Craven**

USA

**Chad Creighton**

USA

**Julie Cridland**

USA

**Daniel Croll**

Switzerland

**Cristina Crosatti**

Italy

**Daniel Crouch**

UK

**Cristiana Crucueanu**

Canada

**Árpád Csernetics**

Hungary

**Miklos Csuros**

Canada

**Suresh Cuddapah**

USA

**Argelia Cuenca**

Denmark

**Dan Cullen**

USA

**Brian Cummings**

USA

**Julie Cunningham**

Italy

**Christina Cuomo**

USA

**Bruce Curtis**

Canada

**Bob Cushman**

USA

**Sally Cutler**

UK

**Nunzio D’Agostino**

Italy

**Xinbin Dai**

USA

**Denise Daley**

USA

**Tim Dallman**

UK

**Rami Dalloul**

USA

**Brian Dalrymple**

Australia

**Annette Damert**

Romania

**Thomas Dandekar**

Germany

**Alberto Danielli**

Italy

**Charles Daniels**

USA

**Roy Danzmann**

Canada

**Wanderson Darocha**

Brazil

**Sandip Das**

India

**Sabyasachi Das**

USA

**Pranab Das**

India

**Malay Das**

India

**George Daston**

USA

**Manish Datt**

India

**Xavier Daura**

Spain

**Giuseppe D’Auria**

Spain

**Jean-Philippe David**

France

**William Davidson**

Canada

**Lance Davidson**

USA

**Brian Davis**

USA

**Thomas Davis**

USA

**Richard Davis**

USA

**Michael Day**

USA

**Sergio De Almeida**

Portugal

**Andre De Almeida**

Portugal

**Marcos De Donato**

Venezuela

**Dirk De Graaf**

Belgium

**Teresa De Kievit**

Canada

**Jose De La Fuente**

Spain

**Fernando Pardo-Manuel De Villena**

USA

**Elzo De Wit**

Netherlands

**Wendy Dean**

UK

**Robert Debuchy**

France

**L Dehmelt**

Germany

**Prescott Deininger**

USA

**Luiz Eduardo Del Bem**

USA

**Christian Delannoy**

UK

**Erick Denamur**

France

**Xiangyu Deng**

USA

**David Denlinger**

USA

**Stuart Dennis**

UK

**Christopher Deppmann**

USA

**Keith Derbyshire**

USA

**Thomas Derrien**

USA

**David Des Marais**

USA

**Philippe Deschamps**

France

**Mohanish Deshmukh**

USA

**Charles Desmarchelier**

France

**Christophe Dessimoz**

USA

**Jeremy Dettman**

Canada

**Robert Devlin**

Canada

**Ralph Dewey**

USA

**Sanghamitra Dey**

India

**Braham Dhillon**

Canada

**Sourabh Dhingra**

USA

**Barbara Di Camillo**

Italy

**Pier Paolo Di Nocera**

Italy

**Valentine Di Pietro**

UK

**Graciela Dias**

Brazil

**Juan Antonio Diaz Pendon**

Spain

**Alexander Diehl**

USA

**Dzung Diep**

Norway

**Dean Diepeveen**

Australia

**Christoph Dieterich**

Germany

**Johannes Dijkstra**

Japan

**Anna Dilger**

USA

**George Dimopoulos**

USA

**Scott Dindot**

USA

**Randy Dinkins**

USA

**Elisabeth Dirlewanger**

France

**Ottmar Distl**

Germany

**Christina Dixelius**

Sweden

**Rekha Dixit**

India

**Brian Dixon**

Canada

**Peter Dodds**

Australia

**Ken Dodds**

New Zealand

**Chris Doe**

USA

**Walter Doerfler**

Germany

**Pavel Dolezal**

Czech Republic

**Orlando Dominguez**

Spain

**Xavier Donadeu**

UK

**Dong Dong**

China

**Joaquin Dopazo**

Spain

**Stefanie Doppler**

Germany

**Oliver Dorigo**

USA

**Brigitte Dorner**

Germany

**Steve Dorus**

USA

**Jerome Doyen**

France

**Peter Dracatos**

Australia

**Michel Drancourt**

France

**Bruce Draper**

USA

**Dayna Dreger**

USA

**Arnold J M Driessen**

Netherlands

**Jenny Drnevich**

USA

**Todd Druley**

USA

**Suzanne Drury**

UK

**Clive D’Santos**

UK

**Ngoc-Hien Du**

Switzerland

**Ruofei Du**

USA

**Xiongming Du**

China

**Chunguang Du**

USA

**Xin Du**

Australia

**Peicheng Du**

USA

**Fang Du**

USA

**Marcio Duarte**

Brazil

**Edward Dudley**

USA

**Aimee Dudley**

USA

**Alexander Dudnikov**

Russian Federation

**Bojan Duduk**

Serbia

**Bernard Dumas**

France

**Ian Dunn**

UK

**Julie Dunning Hotopp**

USA

**Anne Dupressoir**

France

**D Durnford**

Canada

**Olivier Duron**

France

**Chitra Dutta**

India

**Fabien Duveau**

USA

**Sophie Eaglen**

Netherlands

**Esmaeil Ebrahimie**

Australia

**Viviana Echenique**

Argentina

**John-Sebastian Eden**

Australia

**Rolf Edvardsen**

Norway

**Todd Edwards**

USA

**Alexis Edwards**

USA

**David B. Edwards**

Australia

**Dave Edwards**

Australia

**Ian Ehrenreich**

USA

**Ludwig Eichinger**

Germany

**Laura E Eierman**

USA

**Kenneth Eilertsen**

USA

**Ralf Einspanier**

Germany

**Jose M. Eirin-Lopez**

USA

**Rajan Elanchezhian**

UK

**Vahap Eldem**

Turkey

**Marek Elias**

Czech Republic

**Abigail Elizur**

Australia

**Philip Elks**

UK

**Justine Ellis**

Australia

**Marc Eloit**

France

**Maurice Elphick**

UK

**Abdelaleim Elsayed**

Egypt

**Christine Elsik**

USA

**Jessica Elswood**

USA

**Terry Elton**

USA

**Bert Ely**

USA

**Francesco Emanuelli**

Italy

**Joanne Emerson**

USA

**Aidan Emery**

UK

**Frank Emmert-Streib**

Finland

**Scott Emrich**

USA

**Toshinori Endo**

Japan

**Lel Eory**

UK

**Mark Eppinger**

USA

**Mustafa Erayman**

Turkey

**Ionas Erb**

Spain

**Malena Erbe**

Germany

**Li Erchao**

China

**Maria Raffaella Ercolano**

Italy

**Beitz Eric**

Germany

**Carl Ernst**

Canada

**Helga Ertesvåg**

Norway

**Karen Escheverri**

USA

**Carolina Escobar**

Spain

**Luca Espen**

Italy

**Karim Essani**

USA

**Jeffrey J. Essner**

USA

**Thijs Ettema**

Sweden

**Timothy Evans**

USA

**Jared Evans**

USA

**Adam Ewing**

Australia

**Rob Ewing**

UK

**David Eyre**

UK

**Sean Fair**

Australia

**Bryce Falk**

USA

**Chuanzhu Fan**

USA

**Guoqiang Fan**

China

**Jiaqin Fan**

China

**Longjiang Fan**

China

**Gang Fang**

USA

**Fang Fang**

USA

**Wanping Fang**

China

**Weiguo Fang**

China

**Hui Fang**

USA

**Charles Farber**

USA

**Danielle Faria**

Brazil

**Naomi Fast**

USA

**Shadma Fatima**

USA

**Sebastien Faucher**

Canada

**Guido Favia**

Italy

**Maria Federico**

Chile

**Alexei Fedorov**

USA

**Kenneth Feldmann**

USA

**John Fellers**

USA

**Sarah-Maria Fendt**

Belgium

**Brett Ferguson**

Australia

**Francisca Fernandez-Pinas**

Spain

**Manuela Ferracin**

Italy

**Alberto Ferrarini**

Italy

**Paulo Ferreira**

Brazil

**Sergio Ferrero**

Italy

**Martin Ferris**

USA

**James Ferry**

USA

**David Fewer**

Finland

**Stephen Ficklin**

USA

**Daniele Filiault**

USA

**Sergei Filichkin**

USA

**Ertugrul Filiz**

Turkey

**Findley R. Finseth**

USA

**Stefano Fiorucci**

Italy

**Nurit Firon**

Israel

**Peter Fischer**

USA

**Derek Fisher**

USA

**Carolyn Fitzsimmons**

Canada

**Anthony Fiumera**

USA

**Lex Flagel**

USA

**James Flanagan**

UK

**Jean Fleming**

New Zealand

**Damarius Fleming**

USA

**Krzysztof Flisikowski**

Germany

**Lucile Floeter-Winter**

Brazil

**Mario Flores**

USA

**Elena Flowers**

USA

**Brent Fogel**

USA

**Hernan Folco**

USA

**Lasse Folkersen**

Denmark

**Vincent Fondong**

USA

**Nuno Fonseca**

UK

**Luca Fontanesi**

Italy

**Roger Foo**

UK

**Anja Forche**

USA

**Christopher Ford**

Australia

**Alex Ford**

UK

**Thierry Forné**

France

**Konrad Ulrich Förstner**

Germany

**Jamie S Foster**

USA

**Elisabeth Fournier**

France

**Claire Fourrey**

France

**Betsy Foxman**

USA

**Mirko Francesconi**

Spain

**Matthew Francis**

Sweden

**Michael Francki**

Australia

**Susanne Franssen**

Austria

**Lena Fraser**

New Zealand

**Sebastian Fraune**

Germany

**Merete Fredholm**

Denmark

**Jules Freeman**

Australia

**Loreta Freitas**

Brazil

**Laure Fresard**

USA

**Stephan Frickenhaus**

Germany

**Haya Friedman**

Israel

**Adriana Fróes**

Brazil

**James Fry**

USA

**Yong-Bi Fu**

Canada

**Zhengwei Fu**

China

**Yunxin Fu**

USA

**Marc Fuchs**

USA

**Bernard Fuchs**

Germany

**Hodaka Fujii**

Japan

**Akihiro Fujimoto**

Japan

**Shigeki Fujiwara**

Japan

**Kenji Fukunaga**

Japan

**Atsushi Fukushima**

Japan

**Li Fuli**

China

**Kevin Fuller**

USA

**Barbara Funnell**

Canada

**Terrence Furey**

USA

**Daniela Furlan**

Italy

**Vincenzina Fusco**

Italy

**Matthias Futschik**

Portugal

**John Fyfe**

USA

**Humberto Gajardo**

Chile

**Prabu Gajjeraman**

India

**J.G. Gall**

USA

**Giulio Galla**

Italy

**Jason Gallant**

USA

**Cristian Gallardo-Escárate**

Chile

**Irene Gallego Romero**

USA

**Lina Gallego‐Giraldo**

USA

**Daniel Gallie**

USA

**Nicolas Galtier**

France

**Gennaro Gambardella**

Italy

**Anna Gambin**

Poland

**Susheng Gan**

USA

**Laxman Gangwani**

USA

**Timothy Gant**

UK

**Roman Ganta**

USA

**Zexia Gao**

China

**Meiying Gao**

China

**Jianwei Gao**

China

**Nigel Gapper**

USA

**Enrico Garattini**

Italy

**Jessica Garb**

USA

**Dominique Garcia**

France

**Maria Jesus Garcia**

Spain

**Daniel Garcia De La Serrana**

UK

**Jordi Garcia-Fernandez**

Spain

**Jordi Garcia-Mas**

Spain

**Armando García-Ortega**

USA

**Jose Luis Garcia-Perez**

Spain

**Daniel Garcia-Seco**

Spain

**M. Pilar Garcillán-Barcia**

Spain

**Susan Gardiner**

New Zealand

**Donald Gardiner**

Australia

**Jennifer Gardy**

Canada

**Fabio Gasparini**

Italy

**Giuliano Gasperi**

Italy

**Charles Gasser**

USA

**Sophie Gaudriault**

France

**Joël Gautron**

France

**Florian Geay**

Belgium

**Christiane Gebhardt**

Germany

**Danny Geelen**

Belgium

**Scott Geib**

USA

**David Geiser**

USA

**Thomas Geissmann**

France

**Mikhail Gelfand**

Russian Federation

**Christopher L. Gentile**

USA

**Laurent Gentzbittel**

France

**Marco Gerdol**

Italy

**James Gern**

USA

**Lena Gerwick**

USA

**Olivier Gevaert**

USA

**Pablo Ghiringhelli**

Argentina

**Sujoy Ghosh**

Singapore

**Partho Ghosh**

USA

**Jennifer Gibbons**

USA

**Alicia Gibello**

Spain

**Philip Giffard**

Australia

**Rosario Gil**

Spain

**Clare Gill**

USA

**Steven Gill**

USA

**Michael Gillings**

USA

**Guillermo Giovambattista**

Argentina

**Stephen Giovannoni**

USA

**John Glass**

USA

**Galina Glazko**

USA

**Daniel Glez-Peña**

Spain

**Gernot Gloeckner**

Germany

**Chris Glover**

New Zealand

**Anna Godhe**

Sweden

**Laurence Godiard**

France

**Laurence Godiard**

USA

**Aaron Golden**

USA

**Paul Gollnick**

USA

**Jorge Enrique Gomez-Marin**

Colombia

**Ludvik Gomulski**

Italy

**Yoichi Gondo**

Japan

**Cedric Gondro**

Australia

**Chengliang Gong**

China

**Binsheng Gong**

USA

**Josefa Gonzalez**

Spain

**Am González-Tizón**

Spain

**Hani Goodarzi**

USA

**Uri Gophna**

Israel

**Mian-Chee Gor**

Australia

**Stephen Gordon**

Ireland

**Tobias Goris**

Germany

**Gregor Gorjanc**

UK

**Sara Gosline**

USA

**Toni Gossmann**

UK

**Shin Goto**

Japan

**Jinying Gou**

China

**Benjamin Gourion**

France

**Revathi Govind**

USA

**Nermin Gozukirmizi**

Turkey

**Manfred Grabherr**

Sweden

**Mikhail A. Grachev**

Russian Federation

**Laurie Graham**

Canada

**Neil Graham**

UK

**Stella Grando**

Italy

**Gregor Grass**

Germany

**Sonja Grath**

Germany

**Christian Gray**

UK

**Lesley Greene**

USA

**Michael Greenwood**

UK

**Gustavo Gregoracci**

Brazil

**Peter Gresshoff**

Australia

**Etienne Grienenberger**

USA

**Anthony Griffiths**

USA

**Jérôme Grimplet**

Spain

**Delphine Grivet**

Spain

**Astrid Groot**

Netherlands

**Christine Große-Brinkhaus**

Germany

**Ewald Grosse-Wilde**

Germany

**Deborah Grove**

USA

**Andy Groves**

USA

**Christina Grozinger**

USA

**Jens Gruber**

Germany

**Christoph Grunau**

France

**Amy Grunden**

USA

**Angelika Gründling**

UK

**Frank Grutzner**

Australia

**Dariusz Grzebelus**

Poland

**Lianfeng Gu**

China

**Jianying Gu**

USA

**Zuang Gu**

Germany

**Le Luo Guan**

Canada

**Ernesto Guccione**

Singapore

**Yannick Gueguen**

France

**Michael J. Guertin**

USA

**Muriel Gugger**

France

**Alice Guidot**

France

**Andre Guillouzo**

France

**Ulrich Güldener**

Germany

**Patrick Gulick**

Canada

**Ozan Gundogdu**

UK

**Arthur Günzl**

USA

**Xingli Guo**

China

**Qiaosheng Guo**

China

**Huatao Guo**

USA

**Guangwu Guo**

USA

**Anyuan Guo**

China

**Radhey Gupta**

Canada

**Om Prakash Rakash Gupta**

India

**Tugba Gurkok**

Turkey

**Tony Gutschner**

USA

**Rene Guyomard**

France

**Frank Guzman**

USA

**Prasad Gyaneshwar**

USA

**Hubertus Haas**

Austria

**Stefan Haas**

Germany

**Wilhelm Haas**

USA

**Kelly Haas**

USA

**Brian Haas**

USA

**Brett Haberstick**

USA

**Orçun Haçariz**

Turkey

**Abderrahman Hachani**

UK

**Hubert Hackl**

Austria

**Darren Hagen**

USA

**Daisuke Hagiwara**

Japan

**Daniel Hahn**

USA

**Regine Hakenbeck**

Germany

**John Haley**

USA

**Marc Halfon**

USA

**Raphael Hamawaki**

USA

**Bjoern Hamberger**

Denmark

**Gabriel Hamer**

USA

**Ruth Hamill**

Ireland

**Jochen Hampe**

Germany

**Kristin Hamre**

Norway

**Tianfu Han**

China

**Gang Han**

USA

**Yingpeng Han**

China

**Xiaoxu Han**

China

**James Hane**

Australia

**Lauren Hanna**

USA

**Sridhar Hannenhalli**

USA

**Eilis Hannon**

UK

**Dave Hansen**

Canada

**Peter Hansen**

USA

**Ola Hansson**

Sweden

**Weilong Hao**

USA

**Yuichiro Hara**

Japan

**Anna-Pavlina Haramis**

Netherlands

**Omar Harb**

USA

**Matthias Harbers**

Japan

**Josée Harel**

Canada

**Sajeet Haridas**

USA

**Paul Harrison**

Canada

**Robert L. Harrison**

USA

**Ewan Harrison**

UK

**John Hartung**

USA

**Zahra Hasan**

Pakistan

**C Hash**

India

**Yehudit Hasin**

USA

**Syed Shah Hassan**

Brazil

**Musa Hassan**

UK

**Martin Hasselmann**

Germany

**Bernhard Haubold**

Germany

**Aage Haugen**

Norway

**Alan Hauser**

USA

**Thomas Haverkamp**

Norway

**Qing He**

USA

**Kanglai He**

China

**Zhisong He**

China

**Guangyuan He**

China

**Jiang He**

USA

**Xiang He**

China

**Richard Head**

USA

**Simon Heath**

Spain

**Dieter Heermann**

Germany

**Jan-Hendrik Hehemann**

Germany

**Jayne Hehir-Kwa**

Netherlands

**Marzieh Heidaritabar**

Netherlands

**Stefan Heinl**

Austria

**Emanuel Heitlinger**

Germany

**Gerwin Heller**

Austria

**Richard Helm**

USA

**Nicholas Heng**

New Zealand

**Isabelle Henry**

USA

**Shelley Hepworth**

Canada

**Allen Herbst**

Canada

**Dominique Hermier**

France

**Felipe Hernandes Coutinho**

Brazil

**Pilar Hernandez**

Spain

**Carlos Hernandez-Garcia**

USA

**Alfredo Herrera-Estrella**

Mexico

**Klemens Hertel**

USA

**Ronna Hertzano**

USA

**Moritz Hess**

Germany

**Ahlke Heydemann**

USA

**Andreas Heyland**

Canada

**Paul Hickner**

USA

**Julie Hicks**

USA

**Ari Mikko Hietala**

Norway

**Paul Higgins**

USA

**James David Higgins**

UK

**Chris Hill**

USA

**Catherine Hill**

USA

**N. Luisa Hiller**

USA

**Luisa Hiller**

USA

**Michael Hiller**

Germany

**Heinz Himmelbauer**

Spain

**Yasuyo Himuro**

Japan

**Anke Hinney**

Germany

**Akiya Hino**

Japan

**Kiichi Hirota**

Japan

**Hiroshi Hisano**

Japan

**Mahmut Can Hiz**

Turkey

**Julie Hofer**

India

**Federico Guillermo Hoffmann**

USA

**Monica Hofte**

Belgium

**Saskia Hogenhout**

UK

**Matthew Holden**

UK

**Brenden Holland**

USA

**Michelle Holland**

UK

**Mads Vilhelm Hollegaard**

Denmark

**Mads Hollegaard**

Denmark

**Clare Holleley**

Australia

**Kathryn Holt**

Australia

**Christina Holzapfel**

USA

**Gary Hon**

USA

**Shozo Honda**

USA

**Kyung-Won Hong**

South Korea

**Hongkun Hongkun Zheng**

China

**Tasuku Honjio**

Japan

**Sharon Hook**

Australia

**Katarzyna Hooks**

France

**John Hopkins**

UK

**Tiago Hori**

Canada

**Paul Horrocks**

UK

**Julia Horsfield**

New Zealand

**David Horvath**

USA

**Benjamin A. Horwitz**

Israel

**Xilin Hou**

China

**Ross Houston**

UK

**Susan Howard**

USA

**Kerstin Howe**

UK

**Tzung-Fu Hsieh**

USA

**Y. I. Hsing**

Taiwan

**Juchun Hsu**

Taiwan

**Chun-Nan Hsu**

USA

**Pei-Yin Hsu**

USA

**Zhihong Hu**

China

**Patrick John Hu**

USA

**Fuquan Hu**

China

**Yang Huaiyi**

China

**Yadong Huan**

USA

**Lili Huang**

China

**Fang Huang**

China

**Wen Huang**

USA

**Jun Huang**

China

**Yen-Tsung Huang**

USA

**Hao-Jen Huang**

Taiwan

**Sanwen Huang**

China

**Di Huang**

USA

**Simon Hubbard**

UK

**Matthew Hudson**

USA

**Nicholas Hudson**

Australia

**Laura Hug**

USA

**Thomas Hughes**

UK

**Jerome Hui**

Hong Kong

**Amanda Hulse-Kemp**

USA

**Marcel Hulst**

Netherlands

**Amanda Hummon**

USA

**Jui-Hung Hung**

Taiwan

**Patricia Hunt**

USA

**Peter Hunt**

Australia

**Wayne Hunter**

USA

**Klaus Huse**

Germany

**Stephan Hutter**

Germany

**Ann Kathrin Huylmans**

Germany

**Torgeir R. Hvidsten**

Norway

**Pung-Pung Hwang**

Taiwan

**Doug Hyatt**

USA

**John Hyde**

USA

**Ximena Ibarra-Soria**

UK

**Muhammad Ibrahim**

PAKISTAN

**Kazuo Ichimura**

Japan

**Alexander Idnurm**

Australia

**Kate Ihle**

Panama

**Daisuke Ikeda**

Japan

**Takuya Imamura**

Japan

**Juan Imperial**

Spain

**Diane Inglis**

USA

**Hanne Ingmer**

Denmark

**Takao Inoue**

Singapore

**Thomas Ioerger**

USA

**Francesco Iorio**

UK

**Muhammad Iqbal**

USA

**Judith Irwin**

UK

**Jane Irwin**

Ireland

**Naveed Ishaque**

Germany

**Maria Islas-Osuna**

Mexico

**Sachiko Isobe**

Japan

**Yasuhiro Ito**

Japan

**Takeshi Itoh**

Japan

**Masayoshi Itoh**

Japan

**Takehiko Itoh**

Japan

**Nikolai Ivanov**

Switzerland

**Mj Izquierdo Rico**

Spain

**Jeanne Jacobs**

New Zealand

**Jonathan Jacobs**

USA

**Annick Jacq**

France

**Guru Jagadeeswaran**

USA

**Dieter Jahn**

Germany

**Sarika Jalan**

India

**Tim James**

USA

**Guilhem Janbon**

France

**James Jancovich**

USA

**Jeroen Jansen**

Netherlands

**Chatchawan Jantasuriyarat**

Thailand

**Erich Jarvis**

USA

**Stephanie Jaubert-Possamai**

France

**Daniel Jeffares**

UK

**Aaron Jeffries**

UK

**Ken Jeffries**

USA

**Shih-Tong Jeng**

Taiwan

**Ronald Jenner**

UK

**Francis Jenney**

USA

**Jae-Pil Jeon**

South Korea

**Aashish Jha**

USA

**Haiyan Jia**

USA

**Hongbo Jiang**

China

**Cong Jiang**

USA

**Dianhua Jiang**

USA

**Haobo Jiang**

USA

**Eva Jimenez-Guri**

Spain

**Xiaoli Jin**

China

**Fulai Jin**

USA

**Vladimir Jiranek**

Australia

**Hanna S. Johannesson**

Sweden

**Gary Johnson**

USA

**Shannon Johnson**

USA

**Tricia Johnson**

New Zealand

**Todd A. Johnson**

Japan

**Paul Johnston**

New Zealand

**Kimberley Johnstone**

Canada

**Julia Jones**

UK

**Christopher Jones**

UK

**David Jones**

Germany

**Richard Jovelin**

Canada

**Antonio Juárez**

Spain

**Howard Judelson**

USA

**Celina Juliano**

USA

**Ki-Hong Jung**

South Korea

**Piyada Juntawong**

Thailand

**Dilafruz Juraeva**

Germany

**Surendra K Chikara**

India

**Kenneth Ka Ho Lee**

China

**Haja Kadarmideen**

Denmark

**Sebastian Kadener**

Israel

**Ngugi Eliud Kahiu**

KENYA

**Christoph Kaleta**

Germany

**Vipin Kalia**

India

**Péter Kaló**

Hungary

**Auinash Kalsotra**

USA

**Kazuo Kamimura**

Japan

**Jan Kammenga**

Netherlands

**Bavesh Davandra Kana**

South Africa

**Ramesh Kandimalla**

USA

**Yu Kang**

China

**Seogchan Kang**

USA

**Le Kang**

China

**Stella Kantartzi**

USA

**Debapratim Kar Chowdhuri**

India

**Rumyana Karlova**

Netherlands

**Robert Karn**

USA

**Timothy Karr**

USA

**Takao Kasuga**

USA

**Surekha Katiyar-Agarwal**

India

**Hirotomo Kato**

Japan

**Leonard Katz**

USA

**Michael Katze**

USA

**Benita Katzenellenbogen**

USA

**Liisa Kauppi**

Finland

**Nagendra Kaushik**

South Korea

**Ibrahim Kavakli**

Turkey

**Yoshihiro Kawahara**

Japan

**Ryouka Kawahara-Miki**

Japan

**Tom Kawula**

USA

**Kemal Kazan**

Australia

**Haig Kazazian**

USA

**Calvin Keeler**

USA

**Christopher Keeling**

Canada

**David Kehoe**

USA

**Jonathan Keith**

Australia

**Samir Kelada**

USA

**Nancy Keller**

USA

**Beat Keller**

Switzerland

**Ben Kelly**

USA

**Scott Kennedy**

USA

**Carole Kerdelhue**

France

**Shashank Keshavmurthy**

Taiwan

**Shashank Keshavmurthy**

China

**Els Keunen**

Belgium

**Hasan Khatib**

USA

**Mehar Khatkar**

Australia

**Abderrahman Khila**

France

**Abderrahman Khila**

Canada

**Penny Kianian**

USA

**Arash Kianianmomeni**

Germany

**Gustavo Kijak**

USA

**Eui-Soo Kim**

USA

**Kwan Suk Kim**

South Korea

**Youngchul Kim**

USA

**Yongbaek Kim**

South Korea

**Jeongwoon Kim**

USA

**Kami Kim**

USA

**Il-Man Kim**

USA

**Sanguk Kim**

South Korea

**Kwang-Youn Kim**

USA

**Heui-Soo Kim**

South Korea

**Rebecca Kimball**

USA

**Chava Kimchi-Sarfaty**

USA

**W. Allan King**

Canada

**Graham King**

Australia

**Andrew King**

UK

**Justin Kinney**

USA

**John Kirby**

USA

**Jessica Kissinger**

USA

**Todd Kitten**

USA

**Caghan Kizil**

Germany

**Bernd Klaus**

Germany

**Anita Klein**

USA

**Sharon Kleinschmidt**

Australia

**Peter-Christian Kloehn**

UK

**Pamela Knapp**

USA

**Olaf Kniemeyer**

Germany

**Volker Knoop**

Germany

**Gitte Knudsen**

Denmark

**Ichizo Kobayashi**

Japan

**Takanori Kobayashi**

Japan

**Ahmet Koc**

Turkey

**Hauke Koch**

USA

**Lizette Koekemoer**

South Africa

**Lisette Kogelman**

Denmark

**Tong-Wey Koh**

USA

**Gurjjet Kohli**

Australia

**Satomi Kohno**

USA

**Kenji Kojima**

Japan

**James Koltes**

USA

**Dawn Koltes**

USA

**Reina Komiya**

Japan

**Arun Kommadath**

Canada

**Jan Komorowski**

Sweden

**Fyodor Kondrashov**

Spain

**Xiaoyu Kong**

China

**Byung-Whi Kong**

USA

**Panida Kongsawadworakul**

Thailand

**Dusan Kordis**

SLOVENIA

**Sergey Koren**

USA

**Ian Korf**

USA

**Nicholas Korir**

Kenya

**Abraham Korol**

Israel

**Trond M Kortner**

Norway

**Matthew Kostek**

USA

**Linda Kothera**

USA

**Michail Kotsyfakis**

Czech Republic

**Lazlo Kovacs**

USA

**Ales Kovarik**

Czech Republic

**Christine Kozak**

USA

**Aleksei Krasnov**

Norway

**Jeroen Krijgsveld**

Germany

**Vivek Krishnakumar**

USA

**Anne Krogsdam**

Austria

**Jens Kromer**

Australia

**Lee Kross**

USA

**Ricardo Kruger**

Brazil

**T. Książczyk**

Poland

**Rui Kuang**

USA

**Christian P. Kubicek**

Austria

**Ulrich Kück**

Germany

**Can Kucuk**

Turkey

**Christa Kuehn**

Germany

**Heiner Kuhl**

Germany

**Mary Kuhner**

USA

**Anna Kukekova**

USA

**Yutaro Kumagai**

Germany

**Vivek Kumar**

USA

**Anuj Kumar**

USA

**Rahul Kumar**

India

**Manish Kumar**

India

**Swathi Kumar**

USA

**Ranjeet Kumar**

India

**Nagalingam Kumaran**

Australia

**Yoshinori Kumazawa**

Japan

**Richard Kuo**

UK

**Tetsuo Kushiro**

Japan

**Sebastian Kvist**

USA

**Erika Kwon**

USA

**Dennis Kyle**

USA

**Jessy Labbe**

USA

**Vincent Lacroix**

France

**Jason Ladner**

USA

**Emmanuel Ladoukakis**

Greece

**Astrid Lægreid**

Norway

**Zhongxiong Lai**

China

**John Lai**

Australia

**Rup Lal**

India

**Jorge Lalucat**

Spain

**Carles Lalueza Fox**

Spain

**Hon-Ming Lam**

Hong Kong

**Jonathan Lamarre**

Canada

**Elise Lamont**

USA

**Miriam Land**

USA

**Charles Lang**

USA

**Peter Langfelder**

USA

**Michael T. Lardelli**

Australia

**Denis Larkin**

UK

**André Laroche**

Canada

**Amanda Larracuente**

USA

**Eduardo Larriba**

Spain

**Luis Larrondo**

Chile

**Erica Larschan**

USA

**Lars Allan Larsen**

Denmark

**Jill Larsen**

Australia

**Erik Larsson**

Sweden

**Jessica Lasky-Su**

USA

**Christina Laukaitis**

USA

**Federico Lauro**

Singapore

**Carlo Lazado**

Denmark

**Colin Lazarus**

UK

**Diana Le Duc**

Germany

**Gregoire Le Provost**

France

**Karine Gaelle Le Roch**

USA

**Gael Le Trionnaire**

France

**Gerald Leblanc**

USA

**Julie Leclercq**

France

**Dong-Yup Lee**

Singapore

**Hyunju Lee**

South Korea

**Bo-Young Lee**

South Korea

**Ryan Lee**

USA

**Jong-Hee Lee**

South Korea

**Hyunju Lee**

North Korea

**Sang-Rae Lee**

South Korea

**Chia Lee**

USA

**Patrick Lee**

Hong Kong

**Gung Pyo Lee**

South Korea

**Yong-Hwan Lee**

South Korea

**Adrian Lee**

USA

**Laurent Legendre**

France

**J.P. Legg Legg**

Tanzania, United Republic of

**Liancheng Lei**

China

**Dario Leister**

Germany

**Jose Leitao**

Portugal

**Danielle Lemay**

USA

**Ney Lemke**

Brazil

**Andreas Lengeling**

UK

**Boris Lenhard**

Norway

**Emmanuelle Lerat**

France

**Victor Levitsky**

Russian Federation

**Astrid Lewin**

Germany

**Semen Leyn**

Russian Federation

**Zhengguo Li**

China

**Fei Li**

China

**Shaoqing Li**

China

**Zaiyun Li**

China

**Zheng-Nan Li**

China

**Dongdong Li**

China

**Bailin Li**

USA

**Qingzhang Li**

China

**Hu Li**

China

**Genyi Li**

Canada

**Tingting Li**

China

**Ao Li**

China

**Dawei Li**

USA

**Chenghua Li**

China

**Zongjin Li**

China

**Zenglu Li**

USA

**Leiting Li**

China

**Cong-Jun Li**

USA

**Wenbin Li**

China

**Wei Li**

USA

**Jiale Li**

China

**Yinxin Li**

China

**Jun Li**

USA

**Ling Li**

USA

**Yang Li**

USA

**An-Xing Li**

China

**Mingzhou Li**

China

**Xiang Li**

Canada

**Jianke Li**

China

**Mingfeng Li**

USA

**Ying-Hui Li**

China

**Guojing Li**

China

**Shan Li**

USA

**Jiyuan Li**

China

**Youguo Li**

China

**Qi Li**

China

**Ming Li**

USA

**Chengzhi Liang**

China

**Long Liang**

China

**Dan Liang**

China

**Han Liang**

USA

**Haiying Liang**

USA

**Ben-Yang Liao**

Taiwan

**Marc Libault**

USA

**Jessica Liberles**

USA

**Diego Libkind**

Argentina

**Pablo Librado**

Spain

**Benjamin Liebeskind**

USA

**Nina Sylvia Liland**

Norway

**Mark Liles**

USA

**Yuling Lin**

China

**Ping-Ting Lin**

Taiwan

**Senjie Lin**

USA

**Chao-Hsiung Lin**

Taiwan

**Jer-Young Lin**

USA

**Hsin-Nan Lin**

USA

**Tao Lin**

USA

**Qiong Lin**

China

**Fucheng Lin**

China

**Sylvia Lindberg**

Sweden

**Martin Lindner**

Switzerland

**Kristina Lindström**

Finland

**Dirk Linke**

Norway

**Sten Linnarsson**

Sweden

**Paloma Liras**

Spain

**Gianni Liti**

France

**Qiong (Alison) Liu**

USA

**Fang Liu**

China

**Yi Liu**

USA

**Xiling Liu**

China

**Jianyu Liu**

China

**Shiming Liu**

USA

**Zongrang Liu**

USA

**Qing-Jie Liu**

China

**Bao Liu**

China

**Shao-Lun Liu**

Taiwan

**Yuanfa Liu**

China

**Aizhong Liu**

China

**Wansheng Liu**

USA

**Weifeng Liu**

China

**Chun-Chi Liu**

Taiwan

**Liwang Liu**

China

**Tsunglin Liu**

Taiwan

**Shikai Liu**

USA

**Qi Liu**

China

**Jianguo Liu**

China

**Bingqiang Liu**

China

**Kede Liu**

China

**Yuanlong Liu**

China

**Chun-Ming Liu**

China

**George Liu**

USA

**Huiquan Liu**

China

**Tao Liu**

USA

**Baozhong Liu**

China

**Jinping Liu**

China

**Zhanjiang Liu**

USA

**Jihong Liu**

China

**Yuhe Liu**

USA

**Leyuan Liu**

USA

**Bang Liu**

China

**Kaidong Liu**

China

**Xingzhong Liu**

China

**Jianquan Liu**

China

**Wenxuan Liu**

USA

**Brian Livingston**

USA

**Angela Roberta Lo Piero**

Italy

**K Lochovska**

Czech Republic

**James**

Lok USA

**Chunlin Long**

China

**Julie Long**

USA

**Torey Looft**

USA

**Juan Loor**

USA

**Camilo Lopez**

Colombia

**Gloria Lopez-Castejon**

UK

**Emilia López-Solanilla**

Spain

**Elgion Loreto**

Brazil

**Ping Lou**

USA

**Christos Louis**

Greece

**Ricardo Louro**

Portugal

**John Lovell**

USA

**Victor Loyola-Vargas**

Mexico

**Hong Lu**

USA

**Chang Lu**

USA

**Gang Lu**

China

**Ying Lu**

China

**Bin Lu**

China

**Boxun Lu**

China

**Shanfa Lu**

China

**Andrea Luchetti**

Italy

**Ben Luisi**

USA

**Joan Lunney**

USA

**Katie Lunnon**

UK

**Hongmei Luo**

China

**Peigao Luo**

China

**Minhua Luo**

China

**Claire Lurin**

France

**Matthew Lux**

USA

**Qiang Ma**

USA

**Bing Ma**

USA

**Chuang Ma**

China

**Qibin Ma**

China

**Kesen Ma**

Canada

**Carol Macarthur**

USA

**Jiri Macas**

Czech Republic

**Robin Macdiarmid**

New Zealand

**Grant Macgregor**

USA

**Astrid Mach-Aigner**

Austria

**Gustavo Macintosh**

USA

**Simon Mackenzie**

UK

**Andrew Maclean**

USA

**Matthew Macmanes**

USA

**Stephen Madden**

Ireland

**P Madesis**

Greece

**Andreas Madlung**

USA

**Ole Madsen**

Netherlands

**Pascal Maeser**

Switzerland

**Joao Pedro Magalhaes**

UK

**Zenaida Magbanua**

USA

**David Magee**

USA

**Padmanabhan Mahadevan**

USA

**Samuel Maheswaran**

USA

**Martine Maibeche**

France

**Thomas Mailund**

Denmark

**Irina Makarevitch**

USA

**Vsevolod Makeev**

Russian Federation

**Miia Mäkelä**

Finland

**Yuko Makita**

Japan

**Giulia Malacarne**

Italy

**Ryszard Maleszka**

Australia

**Justin Malin**

USA

**James Mallet**

USA

**Ann-Marie Mallon**

UK

**Ilgar Mamedov**

Russian Federation

**Cyril Mamotte**

USA

**David Managadze**

USA

**John Manak**

USA

**Franciele Manboni**

Brazil

**Kranthi Kiran Mandadi**

USA

**Abul Kalam Mandal**

India

**Serghei Mangul**

USA

**Chaysavanh Manichanh**

Spain

**Shuhei Mano**

Japan

**Ben Mans**

South Africa

**Nitin Mantri**

Australia

**Maximino Manzanera**

Spain

**Yunxiang Mao**

China

**Long Mao**

China

**Francismar Marcelino-Guimaraes**

Brazil

**Sergio Marchini**

Italy

**Antonio Marco**

UK

**Gemma Marfany**

Spain

**M. David Marks**

USA

**Fabio Marroni**

Italy

**René Marsano**

Italy

**Tobias Marschall**

Germany

**Frédéric Marsolais**

Canada

**Denise Marston**

UK

**Eric Martens**

USA

**Christina Marth**

Australia

**Paul Martin**

USA

**Sam Martin**

UK

**Samuel Martin**

UK

**Juán Francisco Martín**

Spain

**Federico Martinelli**

Italy

**Manuel Martinez**

Spain

**Esperanza Martinez-Romero**

Mexico

**Iñigo Martínez-Solano**

Portugal

**Natalia Martinkova**

Czech Republic

**Cesar Martins**

Brazil

**Jose Ignacio Martin-Subero**

Spain

**Isabelle Martin-Verstraete**

France

**Marc A. Marti-Renom**

Spain

**Philippe Marullo**

France

**Shinichiro Maruyama**

Japan

**Manja Marz**

Germany

**Cristina Marzachi**

Italy

**Martin Mascher**

Germany

**Annaliese Mason**

Australia

**Wilbert Mason**

USA

**Anna Maria Mastrangelo**

Italy

**Kostas Mathiopoulos**

Greece

**Scot Matkovich**

USA

**Fumio Matsuda**

Japan

**Akihiro Matsui**

Japan

**Masatoshi Matsunami**

Japan

**Yoshihiro Matsuoka**

Japan

**Daisuke Matsuoka**

Japan

**Diethard Mattanovich**

Austria

**Matthias Matter**

Switzerland

**Beverley Matthews**

USA

**Keith Matthews**

UK

**Daniel Philippe Matton**

Canada

**Lakshmi Matukumalli**

USA

**Ildiko Matusikova**

Slovakia

**Daniel Matute**

USA

**Julie Maupin-Furlow**

USA

**Gianluigi Mauriello**

Italy

**Dmitri Mavrodi**

USA

**Siela Maximova**

USA

**Andrew G. Mcarthur**

Canada

**Matthew Mccall**

USA

**Sean Mccarthy**

Ireland

**Ryan Mccleary**

Singapore

**Jeanette Mcclintick**

USA

**Bennet Mccomish**

Australia

**Richard Mcculloch**

UK

**Richard Mceachin**

USA

**John Mcewan**

New Zealand

**Karen Mcginnis**

USA

**Alistair Mcgregor**

UK

**Stephanie Mckay**

USA

**Alan Mcnally**

UK

**John Mcpherson**

Canada

**Matthew Mead**

USA

**Marnix Medema**

Netherlands

**Joaquin Medina**

Spain

**Terrence Meehan**

UK

**Richard Meehan**

UK

**Conor Meehan**

Netherlands

**Wout Megchelenbrink**

Netherlands

**Hendrik-Jan Megens**

Netherlands

**Caroline Meharg**

UK

**Chuansheng Mei**

USA

**Johan Meijer**

Sweden

**Annemarie Meijer**

Netherlands

**Pedro Meirelles**

Brazil

**Miriam Meisler**

USA

**Peter Meister**

Switzerland

**Anne-Leila Meistertzheim**

France

**Richard Meitern**

ESTONIA

**Mariane Melo**

USA

**Ana Mendes-Ferreira**

Portugal

**Ralph Menzel**

Germany

**Maria Merino**

Argentina

**Alex Merrick**

USA

**Felix Mesak**

USA

**Camille Meslin**

USA

**Etienne Meyer**

Germany

**Brian Meyer**

Saudi Arabia

**Rouf Mian**

USA

**Xuexia Miao**

China

**Kristin Michel**

USA

**Sumit Middha**

USA

**Satoshi Mikawa**

Japan

**Michel Milinkovitch**

Switzerland

**Tony Millar**

Australia

**Nicholas Miller**

USA

**Timothy Minogue**

USA

**Patrick Minx**

USA

**Hamid Mirzaei**

USA

**Dan Mishmar**

Israel

**D. C. Mishra**

India

**Prashant Misra**

India

**Biswapriya Misra**

USA

**Caterina Missero**

Italy

**Sara Mitchell**

USA

**Makoto Mitsumori**

Japan

**Masatoshi Miyakoshi**

Japan

**Akio Miyao**

Japan

**Yosuke Mizuno**

Japan

**Gerald Mkoji**

Kenya

**Lenha Mobuchon**

France

**Keiichi Mochida**

Japan

**Tomofumi Mochizuki**

Japan

**Thomas Mock**

UK

**Thomas Moen**

Norway

**Perry Moerland**

Netherlands

**Hooman Moghadam**

Norway

**Tapan Kumar Mohanta**

South Korea

**Firdaus Mohd-Raih**

Malaysia

**Sylvain Moineau**

Canada

**Bertrand Mollereau**

France

**Christian Möllers**

Germany

**Antonio Jose Monforte**

Spain

**Celina Montemayor**

USA

**Eugenia E. Montiel**

USA

**Stephen Moore**

Canada

**Stephen Moose**

USA

**Miguel Morales**

USA

**Claire Morandin**

Finland

**Alan Morgan**

UK

**Garry Morgan**

UK

**Ian Morison**

New Zealand

**Cindy Morris**

France

**Paul Morris**

USA

**Ivan Morrison**

UK

**Janna Morrison**

Australia

**John Morrissey**

Ireland

**Jarrett Morrow**

USA

**David Morse**

Canada

**Mirko Moser**

Italy

**Alan Moses**

Canada

**Alexey Moskalev**

Russian Federation

**Amber Mosley**

USA

**Benny Mote**

USA

**Steve Mount**

USA

**Laurence Mouton**

France

**Isabelle Mouyna**

France

**Wellington Muchero**

USA

**Soren Mueller**

Germany

**Carina Farah Mugal**

Sweden

**Raja Mugasimangalam**

India

**Ashutosh Mukherjee**

India

**Rosario Muleo**

Italy

**Peter Mullany**

UK

**Henry Muller**

Austria

**Karel Muller**

Czech Republic

**John Mulley**

UK

**Hetron Munangandu**

Norway

**Andrew Muntean**

USA

**Kylie Munyard**

Australia

**Hélène Muranty**

France

**Florent Murat**

France

**Moses Muraya**

Germany

**Brenda Murdoch**

USA

**Jerome Murienne**

France

**Susumu Muroya**

Japan

**Muhammed Murtaza**

USA

**Alexander Myburg**

South Africa

**Peter Myler**

USA

**James Nagler**

USA

**Gyoungju Nah**

South Korea

**Gyoung Nah**

South Korea

**Samuel Nahashon**

USA

**Amsha Nahid**

Australia

**Kenji Nakahigashi**

Japan

**Kenta Nakai**

Japan

**Nobutaka Nakashima**

Japan

**Yoichiro Nakatani**

Japan

**Takeru Nakazato**

Japan

**Neetha Nanoth Vellichirammal**

USA

**D Napierala**

USA

**Simona Nardozza**

New Zealand

**Krishnamurthy Natarajan**

India

**Sheila Nathan**

Malaysia

**Marina Naval Sanchez**

Australia

**Roy Navarre**

USA

**Katherinne Navarrete**

Chile

**Daniel Neafsey**

USA

**Matthew Neal**

USA

**Nicolas Negre**

France

**Adam Nelson**

USA

**Wataru Nemoto**

Japan

**Chirag Nepal**

Denmark

**Ben Nephew**

USA

**Nicola Neretti**

USA

**Nathalie Nesi**

France

**Matt Neville**

UK

**Peter Newell**

USA

**Chen Siang Ng**

Taiwan

**Pauline Ng**

Singapore

**Ngoc Nham**

USA

**Yingdong Ni**

China

**Francesco Nicassio**

Italy

**Frank Nicholas**

Australia

**Scott Nicholson**

USA

**Brent Nielsen**

USA

**Caroline Nievergelt**

USA

**Rajni Nigam**

USA

**Martha Nigg**

Canada

**Yoshihito Niimura**

Japan

**Anastasia Nikolskaya**

USA

**Maria Nilsson-Janke**

Germany

**Padma Nimmakayala**

USA

**Elina Niño**

USA

**Masahiro Nishihara**

Japan

**Catherine Nock**

Australia

**Susan Noh**

USA

**Tony Nolan**

UK

**Katia Nones**

Australia

**Joakim Norbeck**

Sweden

**Minou Nowrousian**

Germany

**Francis Nunes**

Brazil

**Sanne Nygaard**

Denmark

**Alan O Doherty**

Ireland

**Berl Oakley**

USA

**Sophie Octavia**

Australia

**Duncan Odom**

UK

**Osamu Ogasawara**

Japan

**Lesley Ogilvie**

Germany

**Atsushi Ogura**

Japan

**Dong Oh**

USA

**Kinji Ohno**

Japan

**Yoshiyuki Ohtsubo**

Japan

**Takashi Okamoto**

Japan

**Keiju Okano**

Japan

**Sezer Okay**

Turkey

**Meritxell Oliva**

Spain

**Sarah G Oliveira**

Brazil

**Brian Oliver**

USA

**Baldomero Olivera**

USA

**Steven Olson**

USA

**Ellis O’Neill**

UK

**Atsushi Ono**

Japan

**Charles Opperman**

USA

**Brenda Oppert**

USA

**Laszlo Orban**

Singapore

**Omar Orellan**

Chile

**Alessandro Ori**

Germany

**Renato Orsi**

USA

**Guillermo Ortí**

USA

**Lucy Osborne**

Canada

**Dani Osman**

Lebanon

**Harald Osmundsen**

Norway

**Mike Osta**

Lebanon

**Michael Oster**

Germany

**Jerzy Ostrowski**

Poland

**Orla O’Sullivan**

Ireland

**Donal O’Sullivan**

UK

**Noriko Osumi**

Japan

**Karim Oualkacha**

Canada

**Ken Overturf**

USA

**Bugra Ozer**

Turkey

**Zahide Neslihan Ozturk**

Turkey

**Neslihan Ozturk**

Turkey

**Tim Paape**

Switzerland

**Stephan Pabinger**

Austria

**Eric Pailhoux**

France

**Tiina Pakula**

Finland

**Dasaradhi Palakodeti**

India

**Joana Palha**

Portugal

**Mark Pallen**

UK

**Subba Palli**

USA

**Nicola Palmieri**

Austria

**Yuan-Xiang Pan**

USA

**Yong-Bao Pan**

USA

**Bahman Panahi**

Iran

**Olivier Panaud**

France

**Vera Pancaldi**

Spain

**Shree Pandey**

India

**Akhilesh Pandey**

USA

**Weijun Pang**

China

**R. Papa**

Italy

**Konstantinos Papadimitriou**

Greece

**Argyris Papantonis**

Germany

**Kai Papenfort**

Germany

**Luis Parada**

Argentina

**John Parant**

USA

**Peter Pare**

Canada

**Chandra Pareek**

Poland

**Swarup Parida**

India

**Christian Parisod**

Switzerland

**Wonkeun Park**

USA

**Ji Yeon Park**

South Korea

**Yong-Jin Park**

South Korea

**Chankyu Park**

South Korea

**Heidi Parker**

USA

**Isobel Parkin**

Canada

**Pietro Parma**

Italy

**Tim Parr**

UK

**John Parsch**

Germany

**Kim Parsons**

USA

**Andrew Pask**

Australia

**Maulik R. Patel**

USA

**Prabhu Patil**

India

**Arunava Pattanayak**

India

**Ned Patterson**

USA

**Hubert Pausch**

Germany

**Martin Pavelka**

USA

**Youri Pavlov**

USA

**Hitesh Pawar**

USA

**Antonio Pazos**

Spain

**Stephanie Pearl**

USA

**Gareth Pearson**

Portugal

**Brian Pedersen**

USA

**Joao Pedra**

USA

**Nemo Peeters**

France

**Liming Pei**

USA

**Clement Pellegrin**

France

**Paolo Pelosi**

Italy

**Jose Penades**

UK

**Francisco Penagaricano**

USA

**Cheng Peng**

China

**Zhao Peng**

USA

**Deepak Pental**

India

**Alan Pepper**

USA

**Luisa Pereira**

Portugal

**Omaththage Perera**

USA

**Miguel Perez-Enciso**

Spain

**Marcos Perez-Losada**

USA

**Ernesto Perez-Rueda**

Mexico

**Jaume Perez-Sanchez**

Spain

**Dragan Perovic**

Germany

**Xavier Perret**

Switzerland

**Sharyn Perry**

USA

**Sudeep Perumbakkam**

USA

**Frank Pessler**

Germany

**Eric Peyretaillade**

France

**Gerd Pfeifer**

USA

**Friedhelm Pfeiffer**

Germany

**Adam Phillippy**

USA

**Amornrat Phongdara**

Thailand

**Pietro Piffanelli**

Italy

**Stefania Pilati**

Italy

**Manoj Pillai**

USA

**Smitha Pillai**

Swaziland

**Manoj Pillay**

USA

**Bo Ping**

China

**Ye Ping**

USA

**Danillo Pinhal**

Brazil

**J. Chris Pires**

USA

**Cary Pirone**

USA

**Gina Pistol**

Romania

**Frederique Pitel**

France

**Jason Pitts**

USA

**Pothitos Pitychoutis**

USA

**David Plachetzki**

USA

**Roy Platt**

USA

**Matthias Platzer**

Germany

**Julio Plaza-Diaz**

Spain

**Amnart Poapolathep**

Thailand

**Lars Podsiadlowski**

Germany

**Monica Poelchau**

USA

**Joe Pogliano**

USA

**Igor Pogribny**

USA

**Eugenia Poliakov**

USA

**Igor Polikarpov**

Brazil

**Kyle Pomraning**

USA

**Teresa Ponce-Noyola**

Mexico

**Siriluck Ponsuksili**

Germany

**Art Poon**

Canada

**Wirulda Pootakham**

USA

**Mihai Pop**

USA

**Michel Popoff**

France

**Eduard Porta-Pardo**

USA

**Emilio Potenza**

Italy

**Nicoletta Potenza**

Italy

**Joël F. Pothier**

Switzerland

**Rodolphe Poupardin**

Czech Republic

**Christine Pourcel**

France

**Michael Povelones**

USA

**Ann Powell**

USA

**Christopher Power**

Canada

**Anne Marie Power**

USA

**Rolf Prade**

USA

**Manoj Prasad**

India

**Grafien Prefontaine**

Canada

**James Prendergast**

UK

**Christopher Previti**

Germany

**Erin Price**

Australia

**Dana Price**

USA

**Alessandro Prigione**

Germany

**Silvas Prince**

USA

**Leighton Pritchard**

UK

**Andrey Prjibelski**

Russian Federation

**Kay Pruefer**

Germany

**Androniki Psifidi**

UK

**Alvaro Puga**

USA

**Pere Puigbo**

USA

**Jose Martin Pujolar**

Italy

**Joanna Pulawska**

Poland

**Peter J Punt**

Netherlands

**Chamindie Punyadeera**

Australia

**Marleen Purcell**

USA

**Guo-Ying Qian**

China

**Wanqiong Qiao**

USA

**Deyou Qiu**

China

**Gao-Feng Qiu**

China

**Shuhao Qiu**

USA

**Jian-Ding Qiu**

China

**Baoli Qiu**

China

**Li Quan**

China

**Youxiong Que**

China

**Luciano Queiroz**

Brazil

**Lina Quesada**

USA

**Edwige Quillet**

France

**John Quinn**

UK

**Matthew R. Willmann**

USA

**Francois Radvanyi**

France

**Sylvain Raffaele**

France

**Saurabh Raghuvanshi**

India

**Greg Ragland**

USA

**Raja Ragupathy**

Canada

**Yasir Rahmatallah**

USA

**Laura Raimondi**

Italy

**Ravi Rajwanshi**

India

**Niaina Rakotosamimanana**

Madagascar

**Ramesh Ramachandran**

USA

**Nirala Ramchiary**

India

**Gopal Ramesh Kumar**

India

**Jose Ramirez**

USA

**Lucía Ramírez**

Spain

**Jorge Ramirez-Prado**

Mexico

**Guillermo Ramis**

Spain

**Clécio Ramos**

Brazil

**Xiaolan Rao**

USA

**David Rasko**

USA

**Sepand Rastegar**

Germany

**Rita Rasteiro**

UK

**Pascal Ratet**

France

**Thenmalarchelvi Rathinavelan**

India

**Thomas Rattei**

Austria

**Terje Raudsepp**

USA

**Dmitry Ravcheev**

Luxembourg

**Rhitoban Raychoudhury**

India

**Ben Raymond**

UK

**Sergey Razin**

Russian Federation

**Timothy Read**

USA

**Catherine Reardon**

USA

**Umesh Reddy**

USA

**James Reecy**

USA

**Kent Reed**

USA

**Robert Reed**

USA

**Hubert Rehrauer**

Switzerland

**Adam Reid**

UK

**Jeff Reid**

USA

**Cavan Reilly**

USA

**Jüri Reimand**

Canada

**Kathleen Rein**

USA

**Adam Reitzel**

USA

**Luke Remage-Healey**

USA

**Simon Renny-Byfield**

USA

**Robert Rentzsch**

Germany

**Martijn Rep**

Netherlands

**Pierre-Yves Rescan**

France

**Ralf Reski**

Germany

**Silvia Restrepo**

Colombia

**Enrique Reynaud**

Mexico

**Mohammad Reza Akbari**

Canada

**Fabio Rezzonico**

Switzerland

**Douglas Rhoads**

USA

**Jose Ribeiro**

USA

**Cintia Ribeiro**

USA

**Scott Rice**

Singapore

**Kelly Rice**

USA

**Vincent Richards**

USA

**Samantha Richardson**

Australia

**Michael K. Richardson**

Netherlands

**Nicole Riddle**

USA

**Laure Ries**

Luxembourg

**Isidore Rigoutsos**

USA

**Monique Rijnkels**

USA

**Laura Rindi**

Italy

**Matthew Rise**

Canada

**Graham Ritchie**

UK

**Alberto Riva**

USA

**Natalia V Rivera**

Sweden

**Renata Rivera-Madrid**

Mexico

**Claude Robert**

Canada

**Richard Roberts**

USA

**Hugh Robertson**

USA

**Alain Robichon**

France

**Stephane Robin**

France

**Charles Robin**

Australia

**Peter Robinson**

Germany

**Marc Robinson-Rechavi**

Switzerland

**James Rocco**

USA

**Dominique Rocha**

France

**Sonia Rocha-Sanchez**

USA

**George Rodakis**

Greece

**Christian Rödelsperger**

Germany

**Raymond Rodgers**

Australia

**Eli Rodgers-Melnick**

USA

**Maria Rosario Rodicio**

Spain

**Francisco Rodriguez-Valera**

Spain

**Stephanie Roessler**

Germany

**Claire Rogel-Gaillard**

France

**Igor Rogozin**

USA

**Sangho Roh**

South Korea

**Jai Rohila**

USA

**Gary Rohrer**

USA

**Antonis Rokas**

USA

**Maria Romay**

USA

**Charles Romieu**

France

**Jonathan Romiguier**

France

**Eric Rondeau**

Canada

**Junkang Rong**

China

**Jun Rong**

Netherlands

**K Ropka-Molik**

Poland

**Abel Rosado**

Canada

**Jason A. Rosenzweig**

USA

**Isabelle Rosinski-Chupin**

France

**Jason Ross**

Australia

**Daniel Ross**

USA

**Jermaine Ross**

USA

**Wolfgang Rottbauer**

Germany

**Jarkko Routtu**

Israel

**Julien Roux**

USA

**Simon Roux**

USA

**Sourav Roy**

USA

**Jason Rudd**

UK

**Oscar Rueda**

UK

**Rosa M. Ruiz-Vazquez**

Spain

**Benedetto Ruperti**

Italy

**Jan Rupp**

Germany

**Eyatan Ruppin**

Israel

**Karsten Ruscher**

Sweden

**Paul Rushton**

USA

**Steve Russell**

UK

**Robert Ryan**

UK

**Marion Ryan**

Ireland

**I Rychlik**

Czech Republic

**Jeffery Saarela**

Canada

**Beatriz Sabater-Munoz**

Ireland

**Chiara Sabatti**

USA

**Giuseppe Saccone**

Italy

**Laurent Sachs**

France

**Ben Sacks**

USA

**Kirsten Sadler**

USA

**Richard Saffery**

Australia

**Boris Sagredo**

Chile

**Sukumar Saha**

USA

**Subhrajit Saha**

USA

**Goutam Sahana**

Denmark

**Mehtap Şahin-Çevik**

Turkey

**Treenut Saithong**

Thailand

**Kuniaki Saito**

Japan

**Katsuharu Saito**

USA

**Kenji Saitoh**

Japan

**Naruya Saitou**

Japan

**Hitoshi Sakakibara**

Japan

**Muhammet Sakiroglu**

Turkey

**Joelle Salazar**

USA

**Elena Salina**

Russian Federation

**Steve Salipante**

USA

**Paulo Salomon**

Brazil

**Maria Salvato**

USA

**Francesco Salvatore**

Italy

**Marco Salvemini**

Italy

**Silvio Salvi**

Italy

**Pedram Samani**

Canada

**Michael Sammeth**

Brazil

**Robert Sammons**

USA

**Federico Sanchez**

Mexico

**David Sanchez-Charles**

Spain

**Alicia Sanchez-Mazas**

Switzerland

**Gabino Sanchez-Perez**

Netherlands

**Yongming Sang**

USA

**Vartul Sangal**

UK

**Walter Sanseverino**

Spain

**Tatiana Santos**

Brazil

**Eidy Santos**

Brazil

**Maria Raquel Santos Carvalho**

Brazil

**Diego Santos-Garcia**

Israel

**Javier Santoyo-Lopez**

UK

**Pinaki Sar**

India

**Gautier Sarah**

France

**Daniel Sargent**

Italy

**Peter Sarkies**

UK

**Derek Sarovich**

Australia

**Elena Sarropoulou**

Greece

**Tetsuhiko Sasaki**

Japan

**Andrea Sass**

Belgium

**Kazuhiro Sato**

Japan

**Yutaka Satou**

Japan

**Yoko Satta**

Japan

**Diane Saunders**

UK

**Thomas Sauter**

Luxembourg

**Christopher Sauvage**

France

**Yuji Sawada**

Japan

**Samir Sawant**

India

**Ruairidh Sawers**

Mexico

**Marcel Schaaf**

Netherlands

**Peter Schaap**

Netherlands

**Roland Schafleitner**

Taiwan

**Leonard Schalkwyk**

UK

**Joost P. Schanstra**

France

**Elke Schaper**

Switzerland

**Michael Scharf**

USA

**Jörn Schattenberg**

Germany

**Flavio Schenkel**

Canada

**Amnon Schlegel**

USA

**Johann Schlesinger**

USA

**Matthias Schlesner**

Germany

**Michael Schlömann**

Germany

**Craig Schluttenhofer**

USA

**Amy Schmid**

USA

**Carl Schmidt**

USA

**Herbert Schmidt**

Germany

**Thomas Schmidt**

Germany

**Stephan Schmitz-Esser**

Austria

**Thomas Schmutzer**

Germany

**Robert Schnabel**

USA

**David Schofield**

USA

**Henrike Scholz**

Germany

**Eric Schott**

USA

**Jonathan Schug**

USA

**Klaus Schughart**

Germany

**Michael Schultze**

UK

**Waltraud Schulze**

Germany

**Frank Schuren**

Netherlands

**Erich Schwarz**

USA

**Marc Scortichini**

Italy

**Marco Scutari**

UK

**Ruth Seal**

UK

**Monica Sebastiana**

Portugal

**Federico Sebastiani**

Italy

**Lisa Seeb**

USA

**Nicola Segata**

Italy

**Vincent Ségura**

France

**Hervé Seitz**

France

**Rajandeep Sekhon**

USA

**Hikaru Seki**

Japan

**Tsuyoshi Sekizuka**

Japan

**Joachim Selbig**

USA

**Herve Seligmann**

France

**Torsten Semmler**

Germany

**Maria Magdalena Senn**

Switzerland

**Kalaiselvi Senthil**

India

**Jeanne Serb**

USA

**Kjell Sergeant**

Luxembourg

**David Serre**

USA

**Margrethe Serres**

USA

**Bertrand Servin**

France

**Jun Sese**

Japan

**Francesco Sestili**

Italy

**Devanshi Seth**

Australia

**Avinash Sethi**

USA

**Joao Setubal**

Brazil

**Alain Sewer**

Switzerland

**Premal Shah**

USA

**Mallikarjun Shakarad**

India

**Ge Shan**

China

**Zhen Shao**

China

**Michael Shapero**

USA

**Arthur Shapiro**

USA

**Igor Sharakhov**

USA

**Thomas Sharkey**

USA

**Anupma Sharma**

USA

**Rajiv Sharma**

UK

**Sushil Sharma**

Netherlands

**Gad Shaulsky**

USA

**Halyna Shcherbata**

Germany

**Andrew Shedlock**

USA

**Martin Sheldon**

UK

**Ekaterina Shelest**

Germany

**Yufeng Shen**

USA

**Jeff Shen**

USA

**Shihua Shen**

China

**Chenjia Shen**

China

**Hui Shen**

USA

**Mao Shengyong**

China

**Daniel Sher**

Israel

**Xinghua Shi**

USA

**Xiaoli Shi**

USA

**Norito Shibata**

Japan

**Lin Shih-Shun**

Taiwan

**Sebastian Shimeld**

UK

**Hiroshi Shimizu**

Japan

**Hideo Shindou**

Japan

**Maki Shirae-Kurabayashi**

Japan

**Kenta Shirasawa**

Japan

**Shin-Han Shiu**

USA

**Eiichi Shoguchi**

Japan

**Tsubasa Shoji**

Japan

**Bin Shuai**

USA

**Smita Shuchi**

USA

**Dorit Shweiki**

Israel

**L. David Sibley**

USA

**Hannah Siddle**

UK

**Stephanie Sidibe Bocs**

France

**Derek Sieburth**

USA

**Mark Siegal**

USA

**Olga Sigalova**

Russian Federation

**Eric Sijbrands**

Netherlands

**Mark Silby**

USA

**Amaro Silva**

Brazil

**Joana Silva**

USA

**Roberto Silva**

Brazil

**Cristina Silvar**

Spain

**David Silver**

Singapore

**Cheolho Sim**

USA

**Richard Simon**

USA

**Carmen Simón-Mateo**

Spain

**Ana Simonovic**

Serbia and Montenegro

**David Sinclair**

USA

**Ratnesh Singh**

USA

**Gajinder Singh**

India

**Dheer Singh**

India

**Sunit Singh**

India

**Pravin Singhal**

USA

**Satrajit Sinha**

USA

**Animesh Sinha**

USA

**Steven Sinkins**

UK

**Sridhar Sivasubbu**

India

**Gottfrid Sjödahl**

Sweden

**Dariusz Skarzynski**

Poland

**Daniel Skelly**

USA

**Patrick Skelly**

USA

**Connor Skennerton**

Australia

**Pontus Skoglund**

USA

**Loren Skow**

USA

**Konstantin G. Skryabin**

Russian Federation

**Jan Slapeta**

Australia

**Jason Slot**

USA

**Michel Slotman**

USA

**Petr Smarda**

Czech Republic

**Age Smilde**

Netherlands

**Nicholas Smirnoff**

UK

**Timothy Smith**

USA

**Hilary Smith**

USA

**Kevin Smith**

USA

**Ryan Smith**

USA

**Theo Smits**

Switzerland

**Davida Smyth**

USA

**Lars Snipen**

Norway

**Darren Soanes**

UK

**Bahar Sogutmaz Ozdemir**

Turkey

**Mahmodul Hasan Sohel**

Germany

**Nicholas Sokol**

USA

**Evgeni Sokurenko**

USA

**Jordi Solana Garcia**

Germany

**Mehmet Somel**

Turkey

**Andreas Sommer**

Austria

**Daniel Sonenshine**

USA

**Fengju Song**

China

**Jiuzhou Song**

USA

**Qijian Song**

USA

**Tad Sonstegard**

USA

**Jorg Soppa**

Germany

**Wim Soppe**

Germany

**Mariana Sottomayor**

Portugal

**Pierre Sourdille**

France

**Filipa Sousa**

Germany

**Pawel Sowinski**

Poland

**Jessica Soyer**

France

**Shanmuga Sozhamannan**

USA

**Manuel Spannagl**

Germany

**Pietro Spanu**

UK

**Doug Speed**

UK

**Juliet Spencer**

USA

**Jana Sperschneider**

Australia

**Andrew Spiers**

UK

**Rebecca Splan**

USA

**Martin Sprick**

Germany

**Patricia Springer**

USA

**Nathan Springer**

USA

**Fabio Squina**

Brazil

**Peerasak Srinives**

Thailand

**Prashant Srivastava**

UK

**Ali Srour**

USA

**Elizabeth Stacy**

USA

**Jason Stajich**

USA

**Kim Stanford**

Canada

**Lawrence Stanton**

Singapore

**Juan Steibel**

USA

**Matthias Steiger**

Austria

**Gary Stein**

USA

**Cristiana Stellato**

Italy

**John Michael Stelling**

USA

**Ioannis Stergiopoulos**

USA

**Ioannis (Yiannis) Stergiopoulos**

USA

**Mark Sterken**

Netherlands

**Marc Stevens**

Switzerland

**John Stevens**

USA

**Karen Stevenson**

UK

**William Stewart**

USA

**Bogdan Stoica**

USA

**Paul Stothard**

Canada

**Timothy Strabala**

New Zealand

**Ralf Stracke**

Germany

**Gale Strasburg**

USA

**Stefan Stricker**

Germany

**Eduard Struys**

Netherlands

**Lisa Stubbs**

USA

**David Studholme**

UK

**Cecil Stushnoff**

USA

**Zhen Su**

China

**Yan-Hua Su**

China

**Victoria Suárez-Ulloa**

USA

**Senthil Subramanian**

USA

**Nikolaus Sucher**

USA

**Peter Sudmant**

USA

**Garret Suen**

USA

**Sebastian Suerbaum**

Germany

**Ruifang Sui**

China

**Zhenghong Sui**

China

**Pavol Sulo**

Slovakia

**Kim Summers**

UK

**Genlou Sun**

Canada

**Junming Sun**

China

**Fengzhu Sun**

USA

**Qixin Sun**

China

**Deqiang Sun**

USA

**Guang Sun**

Canada

**Chuanqing Sun**

China

**Zhongke Sun**

China

**Anitha Sundararajan**

USA

**Ramanjulu Sunkar**

USA

**Granger Sutton**

USA

**Kumar Ray Suvendra**

India

**Yuichiro Suzuki**

USA

**Yutaka Suzuki**

Japan

**Padmapriya Swaminathan**

USA

**Torres Sweeney**

Ireland

**Luc Swevers**

Greece

**William Swindell**

USA

**Jean Swings**

Belgium

**Robert Syme**

Australia

**Karol Szafranski**

Germany

**Ewa Szczuka**

Poland

**David Szuts**

Hungary

**Joshua T Bartoe**

USA

**Peter-Bram ’T Hoen**

Netherlands

**Souvenir Tachado**

USA

**Yuichi Tada**

Japan

**Denis Tagu**

France

**Jibran Tahir**

New Zealand

**Kapil Tahlan**

Canada

**Wataru Takahashi**

Japan

**Fumio Takahashi**

Japan

**Hironori Takasaki**

Belgium

**Naoki Takata**

Japan

**Tomohide Takaya**

Japan

**Haruko Takeda**

Belgium

**Mizuki Takenaka**

Germany

**Rebecca Tallmadge Ingram**

USA

**Dibyendu Talukdar**

India

**Kai Tan**

USA

**Minjia Tan**

China

**Tomoaki Tanaka**

Japan

**Dave Tang**

Japan

**Haixu Tang**

USA

**Kai Tang**

China

**Min Tang**

Japan

**Sithichoke Tangphatsornruang**

Thailand

**Ralph Tanner**

USA

**Bahattin Tanyolac**

Turkey

**Natalia Tapia**

USA

**Lisa Tarantino**

USA

**Mika Tarkka**

Germany

**Phillip Tarr**

USA

**Deirdre Ellen Kathleen Tarr**

USA

**Rebecca Tashiro**

USA

**Age Tats**

Estonia

**Eran Tauber**

UK

**Stefan Taudien**

Germany

**Nektarios Tavernarakis**

Greece

**Frans Tax**

USA

**C Tayade**

Canada

**Marinus Te Pas**

Netherlands

**Scott Tebbutt**

Canada

**Ulrich Technau**

Austria

**Chee-Keng Teh**

Malaysia

**Vladimir Teif**

Germany

**Vladimir Teif**

UK

**Paulo Teixeira**

USA

**Fredj Tekaia**

France

**Janice Telfer**

USA

**Emily Telfer**

New Zealand

**Gianluca Tell**

Italy

**Ross Tellam**

Australia

**Mingxiang Teng**

USA

**Yuanwen Teng**

China

**Stefan Tenzer**

Germany

**Max Teplitski**

USA

**Benno Ter Kuile**

Netherlands

**Valeria Terzi**

Italy

**Andrew Teschendorff**

UK

**Dawit Tesfaye**

Germany

**Alison Testa**

Australia

**Guillaume Tetreau**

USA

**Gianluca Tettamanti**

Italy

**Andreas Teufel**

Germany

**Rita Tewari**

UK

**Ted Thannhauser**

USA

**Louise Thatcher**

Australia

**Johan Thevelein**

Belgium

**Marie-Christine Thibaud**

France

**Danielle Thierry-Mieg**

USA

**Sean Thomas**

USA

**Paul Thomas**

USA

**Milt Thomas**

USA

**Daniela Thomazella**

USA

**Eric Thompson**

Norway

**Cristiane Thompson**

USA

**Andrew Thompson**

Australia

**Joanne Thompson**

UK

**Susan Thomson**

New Zealand

**Michael Thomson**

Philippines

**Jennifer Thomson**

USA

**Michael Thon**

Spain

**Robrecht Thoonen**

USA

**Stefan Thor**

Sweden

**Kevin Thornton**

USA

**Chaoguang Tian**

China

**Zhixi Tian**

China

**Dacheng Tian**

China

**Tianhai Tian**

Australia

**Weidong Tian**

China

**Ruijun Tian**

China

**Michel Tibayrenc**

France

**Denise Tieman**

USA

**Peter Tiffin**

USA

**Zacharewski Tim**

USA

**Gail Timmerman-Vaughan**

New Zealand

**Verlaine Timms**

Australia

**Mbaye Tine**

Germany

**Nick Tinker**

Canada

**Nathan Tintle**

USA

**Ian Tizard**

USA

**Polyana Tizioto**

Brazil

**Raghuvir Tomar**

India

**Eleni Tomazou**

Austria

**Hüseyin Tombuloglu**

Turkey

**Hüseyin Tombuloğlu**

USA

**Craig Tomlinson**

USA

**Yoshinori Tomoyasu**

USA

**Weida Tong**

USA

**Qingchun Tong**

USA

**Ong Chin Tong**

Singapore

**Liu Tong**

China

**Yigang Tong**

China

**Patricia Tonin**

Canada

**Laura Toppino**

Italy

**Kinya Toriyama**

Japan

**Carlos Torroja**

Spain

**Bente Torstensen**

Norway

**Sabine Totemeyer**

UK

**Lam-Son Tran**

Japan

**Yannick Tremblay**

Canada

**Prateek Tripathi**

USA

**Stephen Tristram**

Australia

**Josephine Trott**

USA

**John Trowsdale**

UK

**Guy Tsafnat**

Australia

**Wenchieh Tsai**

Taiwan

**Diogo Tschoeke**

Brazil

**Ka Fai William Tse**

Hong Kong

**George C. Tseng**

USA

**Seiji Tsuge**

Japan

**Stephen Kwok-Wing Tsui**

Hong Kong

**Yoshihiko Tsumura**

Japan

**Takeshi Tsumuraya**

Japan

**Koki Tsuyuzaki**

Japan

**Zhijian (Jake) Tu**

USA

**Shih-Long Tu**

Taiwan

**Bettina Tudzynski**

Germany

**Rolf Turk**

USA

**Serdar Turkarslan**

USA

**Mine Turktas**

Turkey

**Simon Turner**

UK

**Yusuf Tutar**

Turkey

**Jung-Ying Tzeng**

USA

**Wenshyong Tzou**

Taiwan

**Hirohide Uenishi**

Japan

**Anne-Catrin Uhlemann**

USA

**Cirano Ulhoa**

Brazil

**Igor Ulitsky**

USA

**Hidayat Ullah**

Pakistan

**Rainer G. Ulrich**

Germany

**Turgay Unver**

Turkey

**David Ussery**

USA

**David Ussery**

Denmark

**Sergio Uyemura**

Brazil

**Felipe Vaca-Paniagua**

France

**Giampiero Valè**

Italy

**Bruno Valente**

USA

**Alessio Valentini**

Italy

**Nicole Valenzuela**

USA

**Schurdi-Levraud Valerie**

France

**Roger Vallejo**

USA

**Eric Vallender**

USA

**Steven Van Belleghem**

Belgium

**Rianne Van Binsbergen**

Netherlands

**Petra Van Damme**

Belgium

**Jeroen Van De Water**

Australia

**Jan Van Den Abbeele**

Belgium

**Frances Van Dolah**

USA

**Gary Van Domselaar**

Canada

**Freek Van Eeden**

UK

**Frédérique Van Gijsegem**

France

**Sacha Van Hijum**

Netherlands

**Thomas Van Leeuwen**

Belgium

**Benjamin Van Mooy**

USA

**Steve Van Nocker**

USA

**Evert Van Schothorst**

Netherlands

**Marie-Anne Van Sluys**

Brazil

**Paul Vanraden**

USA

**Jens Vanselow**

Germany

**Andre Vanzela**

Brazil

**Miguel Varela**

UK

**Yegor S. Vassetzky**

France

**Nikita Vassetzky**

Russian Federation

**Chantal Vaury**

France

**Heather Veilleux**

Australia

**Paola Venier**

Italy

**Muthusubramanian Venkateshwaran**

USA

**Vittorio Venturi**

Italy

**Ignazio Verde**

Italy

**Sergio Verjovski-Almeida**

Brazil

**Marcel Verk**

Netherlands

**Kenneth Vernick**

France

**Pasqua Veronico**

Italy

**Silvia Vezzulli**

Italy

**Cláudia Vicente**

Portugal

**Renato Vicentini**

Brazil

**Rodrigo Vidal**

Chile

**Maria Lucia Vieira**

Brazil

**Alain Vignal**

France

**Matthieu Vignes**

New Zealand

**Andreas Vilcinskas**

Germany

**Liza Vilela**

Brazil

**Rafael Villa-Angulo**

Mexico

**Sornkanok Vimolmangkang**

Thailand

**Boris Vinatzer**

USA

**Mark Viney**

UK

**Susana Vinga**

Portugal

**Martin Vingron**

Germany

**Marie-Joelle Virolle**

France

**Anna Vivancos**

Spain

**Lila Vodkin**

USA

**Maria Vogelauer**

USA

**Thomas Vogl**

Germany

**Christian Voigt**

Germany

**Jean-Nicolas Volff**

France

**Veronika Von Messling**

Germany

**Bridgett Vonholdt**

USA

**John Vontas**

Greece

**Martin Voskuil**

USA

**Stephen Voss**

USA

**Leslie Vosshall**

USA

**Masaaki Wachi**

Japan

**Simon Waddell**

UK

**Claire Wade**

Australia

**Joseph Wade**

USA

**Johannes Wagener**

Germany

**Anika Eva Wagner**

Germany

**Wolfgang Wagner**

Germany

**Yoshio Wakamatsu**

Japan

**Geoff Waldbieser**

USA

**Seth Walk**

USA

**Charles W Walker**

USA

**Andrew Waller**

UK

**Yizhen Wan**

China

**Jian-Min Wan**

China

**Kai Wang**

USA

**Shichen Wang**

USA

**John Wang**

Taiwan

**Nian Wang**

USA

**Haiyun Wang**

China

**Youping Wang**

China

**Lily Wang**

USA

**Lei Wang**

USA

**Jing Wang**

USA

**Yiming Wang**

Germany

**Ying Wang**

China

**Edwin Wang**

Canada

**Wen Wang**

China

**Zhang Wang**

USA

**Siyun Wang**

Canada

**Fengping Wang**

China

**X. Wang**

USA

**Guo-Dong Wang**

China

**Lei Wang**

China

**Jun Wang**

China

**Jiehua Wang**

China

**Xiaowu Wang**

China

**Lixian Wang**

China

**Liangsheng Wang**

China

**Congli Wang**

China

**Aide Wang**

China

**Xiping Wang**

China

**Zhenping Wang**

China

**Guoqing Wang**

China

**Hao Wang**

USA

**Lijun Wang**

China

**Sibao Wang**

China

**Yingchun Wang**

China

**Chengshu Wang**

China

**Xuchu Wang**

China

**Wenqin Wang**

USA

**Tong Wang**

China

**Huazhong Wang**

China

**Jianbin Wang**

USA

**Jianping Wang**

USA

**Xu Wang**

USA

**Fangjun Wang**

China

**Dierk Wanke**

Germany

**Marilyn Warburton**

USA

**Lucas Ward**

USA

**Melissa Ward**

UK

**Carol Ware**

USA

**Anna Wargelius**

Norway

**Ian Warren**

USA

**Shugo Watabe**

Japan

**Daisuke Watanabe**

Japan

**Robert Waterhouse**

Switzerland

**Bobbie-Jo Webb-Robertson**

USA

**Gareth Weedall**

UK

**Zhu Wei**

China

**Dieter Weichenhan**

Germany

**Rosemarie Weikard**

Germany

**Clifford Weil**

USA

**Shantel Weinsheimer**

Denmark

**Louis Weiss**

USA

**Lauren Weiss**

USA

**Alessandro Weisz**

Italy

**Susan Welburn**

UK

**Joel Ira Weller**

Israel

**Bénédicte Wenden**

France

**Juergen Wendland**

Denmark

**Ching-Feng Weng**

Taiwan

**Jianfeng Weng**

China

**Roman Wenne**

Poland

**Stephen White**

USA

**Michael White**

USA

**Justin Whitehill**

Canada

**David Whitworth**

UK

**Giovanni Widmer**

USA

**George Wiggans**

USA

**Asela Wijeratne**

USA

**Gottfried Wilharm**

Germany

**Jamie Wilkinson**

UK

**James Willey**

USA

**Angela Williams**

Australia

**Peter Williamson**

Australia

**Melissa Wilson Sayres**

USA

**Frederick Wilt**

USA

**Klaus Wimmers**

Germany

**Heidrun Windisch**

Germany

**Thierry Wirth**

France

**Sonia Wirth**

Argentina

**Kamil Witek**

UK

**Edyta Wojtowicz**

Netherlands

**Jochen Wolf**

Sweden

**Yuri Wolf**

USA

**Kyoung Jae Won**

USA

**Hyjung Won**

USA

**Ryan Wong**

USA

**Emily Wong**

UK

**Rasana Wongratanacheewin**

Thailand

**Hyun Goo Woo**

North Korea

**Ian Wood**

UK

**Charles Wood**

USA

**Charles Woodrow**

USA

**Elizabeth Woodward**

USA

**Gert Worheide**

Germany

**Kim Worley**

USA

**Jennifer Wortman**

USA

**Johannes Wöstemeyer**

Germany

**Naomi Wray**

Australia

**Dominic Wright**

Sweden

**Alison Wright**

UK

**Hao Wu**

USA

**Jian Wu**

USA

**Jun Wu**

China

**Zhen Wu**

China

**Wei Wu**

Taiwan

**Ronghu Wu**

USA

**Zhaohui Wu**

USA

**Liang Wu**

China

**Tobias Würschum**

Germany

**Gui-Xian Xia**

China

**Tao Xia**

China

**Rui Xia**

USA

**Ruidong Xiang**

Australia

**Menglan Xiang**

USA

**Shuqi Xiao**

China

**Yun Xiao**

China

**Deyu Xie**

USA

**Jiuyong Xie**

Canada

**Weibo Xie**

China

**Conghua Xie**

China

**Haiping Xin**

China

**Ke Xing**

China

**Ai-Sheng Xiong**

China

**Changjie Xu**

China

**Ruirui Xu**

China

**Xun Xu**

China

**Jinrong Xu**

USA

**Lin Xu**

USA

**Qiang Xu**

China

**Shizong Xu**

USA

**Shuhua Xu**

China

**Jianping Xu**

China

**Yunbi Xu**

China

**Yu Xue**

China

**Xiaoling Xuei**

USA

**Gitanjali Yadav**

India

**Rattan Yadav**

UK

**Nabila Yahiaoui**

France

**Mahmoud Yaish**

OMAN

**Yasunori Yamamoto**

Japan

**Kaneyoshi Yamamoto**

Japan

**Hui Yang**

USA

**Ming-Yu Yang**

Taiwan

**Jinghua Yang**

China

**Ning Yang**

China

**Fengjuan Yang**

China

**Xu Yang Christmas**

Island

**Xiaohe Yang**

USA

**Yao Yang**

USA

**Shihui Yang**

USA

**Qin Yang**

USA

**Jun-Yi Yang**

Taiwan

**Xiaozeng Shawn Yang**

USA

**Pingfang Yang**

China

**Hanchun Yang**

China

**Kentaro Yano**

Japan

**Jianbo Yao**

USA

**Yingyin Yao**

China

**Gloria Yepiz-Plascencia**

Mexico

**Bin Yi**

China

**Xianghua Yi**

China

**Shaowu Yin**

China

**Yanbin Yin**

USA

**Voot Yin**

USA

**Han Yingpeng**

China

**Bauke Ylstra**

Netherlands

**Felipe Yon**

Germany

**Kouki Yonezawa**

Japan

**Dongwan Yoo**

USA

**Yordan Yordanov**

USA

**Hong You**

Australia

**Philip Young**

South Africa

**Terry-Lynn Young**

Canada

**Deyue Yu**

China

**Zhiqiang Yu**

China

**Michael Yu**

USA

**Li Yu**

China

**Jinsheng Yu**

USA

**Mei Yu**

China

**Bin Yu**

USA

**Jun Yu**

China

**Jingjuan Yu**

China

**Jiazheng Yuan**

USA

**Deyi Yuan**

China

**Ling Yuan**

USA

**Tiezheng Yuan**

USA

**Daojun Yuan**

China

**Yihui Yuan**

China

**Wu Yunfeng**

China

**Kei Yura**

Japan

**Vyacheslav Yurchenko**

Czech Republic

**Anita Zamboni**

Italy

**Jesse Zaneveld**

USA

**Yun-Xiang Zang**

South Korea

**Marc Zapatka**

Germany

**Judith Zaugg**

Germany

**Roland Zell**

Germany

**Feng Zeng**

China

**Tao Zeng**

China

**Daniel Zerbino**

UK

**Jixian Zhai**

USA

**Weiwei Zhai**

Singapore

**Yong Zhang**

China

**Xinyue Zhang**

USA

**Linlin Zhang**

USA

**Chunlei Zhang**

China

**Jing Zhang**

USA

**Chuanfu Zhang**

China

**Ziding Zhang**

China

**Qijing Zhang**

USA

**Chi Zhang**

USA

**Xuehong Zhang**

China

**Peng Zhang**

China

**Shiwu Zhang**

China

**Xianghong Zhang**

Switzerland

**Xuegong Zhang**

China

**Chaolin Zhang**

USA

**Jinzhi Zhang**

China

**Chengjun Zhang**

China

**Yanfeng Zhang**

USA

**Wenqing Zhang**

China

**Xinjun Zhang**

USA

**Anding Zhang**

China

**Ze Zhang**

China

**Kui Zhang**

USA

**Jinfa Zhang**

USA

**Yong-Jun Zhang**

China

**Hongbo Zhang**

China

**Shang-Hong Zhang**

China

**Zhaolei Zhang**

Canada

**Runxuan Zhang**

UK

**Shi-Hong Zhang**

China

**Gong Zhang**

China

**Zhenguo Zhang**

USA

**M. Zhang**

China

**Xiaoxia Zhang**

China

**Meiping Zhang**

USA

**Zhe Zhang**

China

**Wenyi Zhang**

China

**Junfei Zhao**

USA

**Patrick Xuechun Zhao**

USA

**Fangqing Zhao**

China

**Jing Zhao**

USA

**Zhongbao Zhao**

China

**Li Zhao**

USA

**Chengchao Zheng**

China

**Lei Zheng**

China

**Deyou Zheng**

USA

**Yun Zheng**

China

**Qi Zheng**

USA

**Dinghai Zheng**

USA

**Boxiong Zhong**

China

**Bojian Zhong**

China

**Ping Zhong**

China

**Yongming Zhou**

China

**Guangyu Zhou**

USA

**Yongcan Zhou**

China

**Feng Zhou**

USA

**Bingsheng Zhou**

China

**X Zhou**

China

**Xiao Zhou**

USA

**Zhi-Hua Zhou**

China

**Hongjun Zhou**

USA

**Yu Zhou**

China

**Chao-Dong Zhu**

China

**Benzhong Zhu**

China

**Shuijin Zhu**

China

**Qian-Hao Zhu**

Australia

**Qiang Zhuge**

China

**Victor Zhurkin**

USA

**Carla Zijlstra**

Netherlands

**Steven A. Zinn**

USA

**Barry Ziola**

Canada

**Xiufen Zou**

China

**Zhen Zou**

China

**Dmitry Zubakov**

Netherlands

**Bo Zuo**

China

**Mario Zurita**

Mexico

**Boris Zybaylov**

USA

